# Coral Reefs on the Edge? Carbon Chemistry on Inshore Reefs of the Great Barrier Reef

**DOI:** 10.1371/journal.pone.0109092

**Published:** 2014-10-08

**Authors:** Sven Uthicke, Miles Furnas, Christian Lønborg

**Affiliations:** Australian Institute of Marine Science, PMB No 3, Townsville, Queensland, Australia; U.S. Geological Survey, United States of America

## Abstract

While increasing atmospheric carbon dioxide (CO_2_) concentration alters global water chemistry (Ocean Acidification; OA), the degree of changes vary on local and regional spatial scales. Inshore fringing coral reefs of the Great Barrier Reef (GBR) are subjected to a variety of local pressures, and some sites may already be marginal habitats for corals. The spatial and temporal variation in directly measured parameters: Total Alkalinity (TA) and dissolved inorganic carbon (DIC) concentration, and derived parameters: partial pressure of CO_2_ (*p*CO_2_); pH and aragonite saturation state (Ω_ar_) were measured at 14 inshore reefs over a two year period in the GBR region. Total Alkalinity varied between 2069 and 2364 µmol kg^−1^ and DIC concentrations ranged from 1846 to 2099 µmol kg^−1^. This resulted in *p*CO_2_ concentrations from 340 to 554 µatm, with higher values during the wet seasons and *p*CO_2_ on inshore reefs distinctly above atmospheric values. However, due to temperature effects, Ω_ar_ was not further reduced in the wet season. Aragonite saturation on inshore reefs was consistently lower and *p*CO_2_ higher than on GBR reefs further offshore. Thermodynamic effects contribute to this, and anthropogenic runoff may also contribute by altering productivity (P), respiration (R) and P/R ratios. Compared to surveys 18 and 30 years ago, *p*CO_2_ on GBR mid- and outer-shelf reefs has risen at the same rate as atmospheric values (∼1.7 µatm yr^−1^) over 30 years. By contrast, values on inshore reefs have increased at 2.5 to 3 times higher rates. Thus, *p*CO_2_ levels on inshore reefs have disproportionately increased compared to atmospheric levels. Our study suggests that inshore GBR reefs are more vulnerable to OA and have less buffering capacity compared to offshore reefs. This may be caused by anthropogenically induced trophic changes in the water column and benthos of inshore reefs subjected to land runoff.

## Introduction

Present day atmospheric carbon dioxide (CO_2_) concentrations are now over 30% higher than the maximum observed in the previous 2 million years [1]. Approximately 28% of this additional CO_2_ is absorbed by the world's oceans [2], [3], leading to lower seawater pH (Ocean Acidification; OA) with reduced carbonate ion concentrations (CO_3_^2−^) and a reduced saturation state (Ω) of calcium carbonate minerals (CaCO_3_). Surface seawater pH has decreased by 0.1 units since pre-industrial times and is predicted to fall by a further 0.3**–**0.5 units in the next 100 years [4]. Large-scale spatial and temporal variations (seasonal, inter-annual) in surface seawater CO_2_ concentrations are known to be caused by biogeochemical and air-sea exchange processes. Knowledge of this variability is critical to understand the current state of the carbon cycle and to predict how the ocean will react to future increases in atmospheric CO_2_ concentration. A recent review indicated that the partial pressure of CO_2_ (*p*CO_2_) in coral reef waters is increasing more rapidly than in the atmosphere, most likely due to other anthropogenic impacts on water quality [5]. Coral reefs in tropical and subtropical regions contribute to the ocean carbon cycle through the processes of photosynthesis, respiration, CaCO_3_ production and dissolution [6], [7]. Coral reef ecosystems are vulnerable to OA and climate change induced ocean warming [8] with a range of effects on the ecosystem and associated biota e.g., [9]–[12]. In particular, increases in oceanic CO_2_ will reduce the aragonite saturation state (Ω_ar_), which decreases the ability of many coral species to produce their carbonate skeletons [10], [13], [14]. As a consequence, future coral reefs may exhibit net-carbonate dissolution as opposed to the net-accretion witnessed today [15].

Because many coral reefs are net-autotrophic, these reefs may have an increased buffering capacity towards OA [16], [17]. Shallow reef flat areas are dominated by respiratory processes at night (increasing CO_2_ in the water) and autotrophic processes during the day (decreasing CO_2_ and thus increasing pH). This can lead to considerable fluctuations of pH, Ω_ar_ and *p*CO_2_ [15], [18], [19].

The Great Barrier Reef (GBR), situated on the NE Australia continental shelf between 9 and 24°S, is the largest contiguous coral reef system in the world. The GBR contains approximately 3,700 individual coral reefs on a shallow shelf with an area close to 250,000 km^2^. Reefs occupy approximately 10% of the shelf area, while most of the remaining shelf is covered with carbonate sediments. The majority of the coral reefs are located on the outer half of the shelf, which is primarily under oceanic influence; however, approximately 20% of reefs lie within 10 km of the coast and are under direct terrestrial influence from freshwater, sediment, nutrient and organic carbon runoff.

Research to date on reef calcification and inorganic carbon dynamics within the GBR system has largely focused upon on-reef processes on mid- and outer-shelf reefs [6], [7], [15], [20]. Relatively little work has been done on the shelf-scale dynamics of inorganic carbon in the GBR system [21], [22] and almost no consideration has been given to the many inshore reefs close to the coast that are under the greatest threat from increases in runoff of sediment, nutrients and pesticides [23]–[25]. The ratio of primary productivity and respiration (P/R) of inshore reefs are often lower than on reefs further from the coast due to decreased light availability associated with greater turbidity inshore [26]–[29]. Because of this, inshore reefs may be less able to buffer rising dissolved inorganic carbon (DIC) by photosynthesis.

Here, we present broad-scale carbon chemistry data from inshore reefs of the GBR, collected six times over two years covering a comprehensive latitudinal range. We tested if there were any persistent regional and seasonal differences between inorganic carbon system parameters in the coastal waters of the GBR. In addition, we compared the carbon chemistry on inshore reefs to a smaller sample set from mid- and outer-shelf reefs and to historical data collected 18 and 30 years ago.

## Materials and Methods

### Sampling design

All work described was covered under a permit obtained from the Great Barrier Reef Marine Park Authority (G12/35236.1).

**Figure 1:**
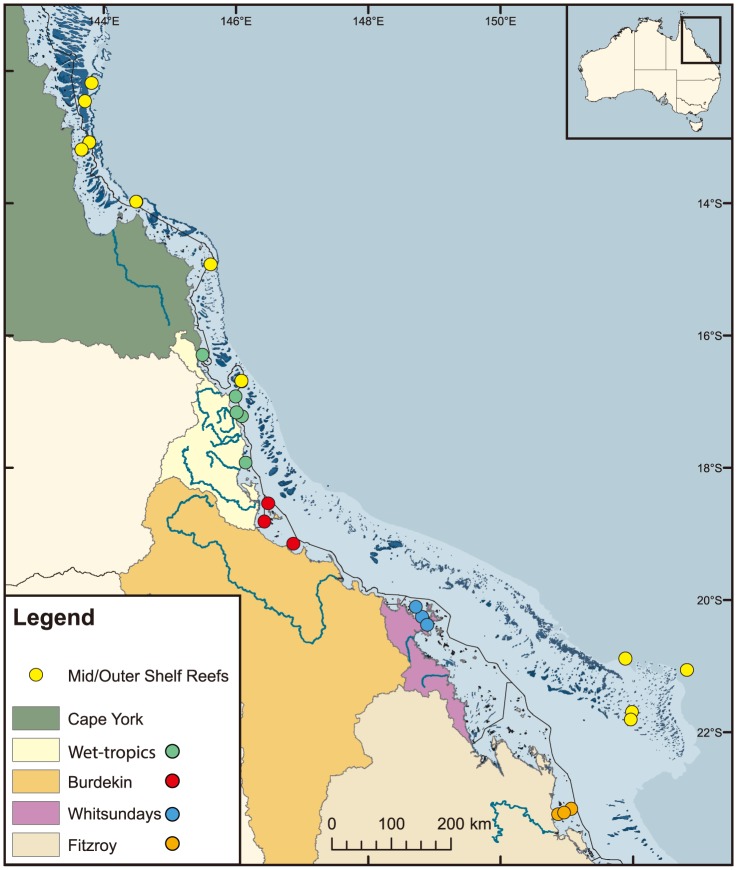
Map showing the sampling stations (•) where surface samples were collected during cruises aboard R/V *Cape Ferguson* over the period September 2011 to November 2013. Inshore monitoring stations, from north to south, are: Snapper Island, Fitzroy Island, High Island, Frankland Island, Dunk Island, Pelorus Island, Pandora Reef, Geoffrey Bay, Double Cone Island, Daydream Island, Pine Island, Humpy and Halfway Islands, Pelican Island, Barren Island, with separate colours representing separate regions as indicated in the legend. Yellow dots indicate mid- and outer-shelf reefs, from north to south: Mantis Reef, Wreck Bay, Fairway Channel, Shipping Channel, Tydeman Reef, Lizard Island, Arlington Reef, Twin Cays, Elusive Reef, Swains Reefs, Inner Swains.

Water sampling for inshore chemical characteristics was carried out at 14 nearshore fringing reefs at islands between 16 and 23° S (Fig. 1). Twelve of the 14 core sites are within 15 km of the mainland and all are directly affected on a seasonal or episodic basis by terrestrial runoff. Sampling at the inshore core reef sites (Visits, n = 6) was conducted at four-monthly intervals over two years (September 2011–June 2013) in the late dry season (September–October), wet season (February) and early dry season (June). The GBR region has a monsoonal climate with most (ca. 60**–**80%) rainfall falling in the January to March period. All samples were collected during the day time. In order to test if values differed between times of the day all samples were grouped into four time brackets (0600**–**0900, 0901**–**1200, 1201**–**1500, and 1501**–**1800) and an overall one factor analysis of variance (ANOVA) was conducted to test if average Total Alkalinity (TA) and dissolved inorganic carbon (DIC) were different between sampling times. This analysis illustrated that there was no significant difference in TA (F_1, 165_ = 0.55, p = 0.6462) or DIC (F_1, 165_ = 1.41, p = 0.2418) values between the four time brackets. The majority (∼65%) of the samples were collected between 0900 and 1500. We therefore concluded that the time of sampling did not bias our spatial or long-term temporal comparisons.

To provide comparison with mid- and outer-shelf reef water we collected additional samples in two GBR regions (the Northern section and the Swains region) on five occasions. Samples from the Northern section were collected in November 2011 (Wreck Bay, Tydeman Reef, and Arlington Reef), June 2012 (Mantis Reef [7 samples] and Lizard Island), and November 2013 (Tydeman Reef and Fairway Channel). Samples from the Swains region were collected in April 2012 (Elusive Reef, Twin Cays Mooring Site, Swains Reefs, and Inner Swains) and September 2013 (Elusive Reef and Swains Reefs). All locations are shown in Fig. 1. Water masses at the outer-shelf sites are primarily influenced by mixing with the oceanic waters of the Coral Sea. For most of its length, the outer-shelf reef matrix is separated from the mainland (and inshore reefs) by an open water body known as the GBR lagoon. South of 15° S, the dominant (non-tidal) water movement on the outer-shelf is to the south under the forcing of the geostrophic pressure gradient of the East Australian Current. Northward surface flows on the shelf may occur during periods of strong SE trade winds. On the inner-shelf, this northward flow driven by SE trade winds is stronger and more persistent. To the north of 15° S, shelf flows are primarily wind-driven.

### Sample collection and analysis

Water samples for analysis of TA and DIC were collected at the 14 core reefs in conjunction with a range of standard oceanographic (temperature and salinity) and water quality (nitrate/nitrite (NO_3_^−^/NO_2_^−^), ammonium (NH_4_^+^), phosphate (PO_4_^3−^), and chlorophyll *a* (Chl *a*)) parameters. The latter parameters are only summarised here to provide a background on the biogeochemical setting of the sites; a more detailed description of these parameters is given elsewhere [30]. At each of the inshore locations, surface (∼1 m water depth) and near-bottom (average depth 9.4 m, 1 SD = 3.1 m) water samples were collected from the R/V *Cape Ferguson* using 10 L Niskin bottles. These open water stations were 0.3**–**2 km from the neighbouring reef. In addition, divers collected water near-bottom (average depth 6.5 m) on the reef slopes of the coral reef at each inshore site.

Duplicate aliquots (250 ml) were carefully drawn from the Niskin bottles for TA and DIC analysis, taking care to avoid bubble formation and minimize headspace. Samples were fixed with 125 µl of saturated HgCl_2_. Samples for TA and DIC were analysed using a VINDTA 3C titrator (Marianda, Germany) at the Australian Institute of Marine Science (AIMS). Alkalinity was determined by acid titration [31] and DIC by acidification and coulometric detection (UIC 5105 Coulometer) of the evolved CO_2_. The VINDTA titrator was calibrated with Certified Reference seawaters (A. G. Dickson, Scripps Institute of Oceanography, Dixon, Batch 106). Raw-data for all TA and DIC samples are given in Table S1 in File S1.

### Historical data

Our data collected in 2011 to 2013 were compared to data collected by Kawahata et al. [22] which were only collected during the dry season (May 1996) and on locations further from the reef than those obtained here. In addition, we obtained historical data from 1982/83 from the AIMS data archive. These were collected by Dr Dave Barnes and colleagues following detailed methods described in [6], [7]. The carbon chemistry calculations from the 1982/83 dataset were based on precision measurements of pH and TA, and those from 1996 on CO_2_ measurements in equilibrator chambers and TA measurements. Although individual methods may vary in their measuring certainty, all are still accepted methods [32] and there is therefore no reason to assume that these data are not comparable. See Table S2 in File S1 for more detailed descriptions and a transcript of the raw data used.

### Data analysis

Carbon chemistry parameters (the partial pressure of CO_2_ [*p*CO_2_], pH on the total scale [pH_Total_]), and the saturation state for aragonite [Ω_ar_]) were calculated using the Excel macro CO2SYS [33], taking salinity and temperature into consideration.

Salinity normalization to a constant salinity is commonly used to correct for differences between source water masses and local effects from evaporation and precipitation on the marine carbon chemistry [34], [35]. We used the method proposed by Friis et al. [34] for TA and DIC, using the annual average salinity during the sampling period of 34.5 and a non-zero freshwater end member [TA_S = 0_ = 309.13 µmol Kg^−1^; DIC_S = 0_ = 288.48 µmol Kg ^−1^].

To separate the seasonal effect of biological processes (B) and temperature (T) on the *p*CO_2_ dynamics, we used the method developed by Takahashi et al. [36] and calculated the effect as:
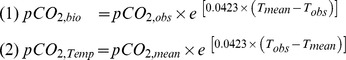
where T is temperature (°C) and the subscripts “mean” and “obs” indicate the annual mean temperatures (reported in Table 1) or *p*CO_2_ for each region and the observed values, respectively. The relative importance of each effect is expressed as the ratio between *p*CO_2, Temp_ and *p*CO_2, bio_ (T/B). A ratio >1 suggests a dominance of temperature effects over biological processes on the *p*CO_2_ dynamics.

**Table 1:** Biological, chemical and physical properties of water samples at the time of collection between 2011 and 2013.

			Sal.	Temp.	Chl *a*	NH_4_^+^	NO_3_^−^/NO_2_^−^	PO_4_^3−^
Area	Season	N		°C	µg l^−1^	µmol kg^−1^	µmol kg^− 1^	µmol kg^– 1^
Wet-tropics	Early dry	20	33.7	23.2	0.33±0.11	0.07±0.04	0.17±0.14	0.11±0.05
	Late dry	20	35.2	25.3	0.28±0.02	0.06±0.05	0.20±0.19	0.11±0.02
	Wet	20	33.7	29.9	0.38±0.17	0.11±0.10	0.18±0.17	0.05±0.02
	All year		34.2	26.1	0.33±0.15	0.08±0.07	0.18±0.16	0.11±0.04
	Amplitude		3.6	8.0	0.75	0.37	0.80	0.22
Burdekin	Early dry	12	34.6	22.5	0.25±0.06	0.07±0.07	0.14±0.09	0.11±0.02
	Late dry	12	35.3	24.7	0.48±0.27	0.11±0.12	0.24±0.22	0.11±0.03
	Wet	12	33.9	30.0	0.34±0.13	0.11±0.07	0.16±0.18	0.05±0.02
	All year		34.6	25.7	0.38±0.22	0.10±0.09	0.18±0.17	0.09±0.04
	Amplitude		3.2	10.0	0.75	0.37	0.76	0.16
Whitsundays	Early dry	12	34.3	22.2	0.45±0.16	0.10±0.05	0.25±0.13	0.15±0.04
	Late dry	12	35.2	23.0	0.41±0.17	0.06±0.04	0.11±0.05	0.14±0.03
	Wet	12	34.8	29.0	0.58±0.11	0.19±0.15	0.26±0.22	0.09±0.03
	All year		34.8	24.7	0.48±0.17	0.13±0.12	0.21±0.16	0.12±0.04
	Amplitude		1.6	8.4	0.59	0.41	0.56	0.16
Fitzroy	Early dry	12	35.1	21.0	0.46±0.28	0.03±0.02	0.11±0.06	0.10±0.06
	Late dry	12	35.6	22.0	0.36±0.42	0.05±0.05	0.16±0.13	0.13±0.05
	Wet	12	33.5	28.0	0.59±0.18	0.11±0.11	0.18±0.22	0.11±0.07
	All year		34.7	23.7	0.47±0.31	0.07±0.08	0.15±0.15	0.12±0.06
	Amplitude		4.6	10.4	1.17	0.28	0.64	0.28
Offshore	Early dry	18	34.9	25.8	0.39±0.18	0.03±0.05	0.12±0.07	0.09±0.04
	Late dry	23	35.4	26.7	0.29±0.23	0.03±0.05	0.13±0.06	0.06±0.02
	Wet		n.d	n.d	n.d	n.d	n.d	n.d
	All year		n.d	n.d	n.d	n.d	n.d	n.d
	Amplitude		1.7	7.2	1.02	0.18	0.31	0.21

Average values for salinity (Sal.), temperature (Temp.), chlorophyll *a* (Chl *a*), ammonium (NH_4_^+^), Nitrate/Nitrite (NO_3_^–^/NO_2_^–^) and phosphate (PO_4_^3−^) and their amplitude (maximum minus minimum level) are shown. Standard deviations are shown for chlorophyll *a* and nutrient data; N: number of samples used to calculate the average.

Salinity-normalized data for TA (TA_S_) and DIC (DIC_S_) were also used to create TA_S_ vs DIC_S_ plots to examine the impact of calcification on the annual changes in the carbon system. This approach follows on from the assumption that net primary production of one mole of organic C reduces DIC by one mole, while calcification reduces TA by two moles and DIC by one mole for each mole of CaCO_3_ precipitated [37]. In systems where calcification is dominating, there should therefore be a linear relationship between DIC and TA with a slope approaching 2.0. The slope of this relationship can be used to calculate the net ecosystem production (NEP) to net ecosystem calcification (NEC) ratio, which is given by the function: (2/slope)-1 [37].

We used mixed model ANOVAs to examine sources of variation in observed levels of TA, DIC, *p*CO_2_, pH_Total_, and Ω_ar_. The fixed main factor “Region” was used to test for differences between the five regions (Wet-tropics, Burdekin, Whitsundays, Fitzroy and offshore). We considered replicate “Islands” as a random nested factor within Regions. To evaluate if samples taken from the reef slopes were different from those collected from the research vessel “Location” was included in the model as a second fixed factor. The main factor “Visit” tested for differences between the six sample periods. With the exception of pH (that is already on a log-scale) all data were log-transformed prior to analysis. Boxplot and residual plots indicated no deviation from ANOVA assumptions for the transformed variables. We tested for correlations between several parameters using Pearson's product moment correlations. To further investigate differences between seasons and inshore *vs* offshore reefs, we conducted a principal component analysis (PCA) with TA, DIC, *p*CO_2_ and Ω_ar_ as carbon chemistry parameters. pH was omitted from this analysis as it is highly correlated with *p*CO_2_. For the comparison of the historical data, we calculated a Bayesian 95% confidence interval for the rate of change based on averages and standard deviations from present day and historical data. This was achieved using Markov Chain Monte Carlo sampling. After a burn-in period of 2000 steps, 5000 steps were sampled and three parallel chains run. These models were also used to calculate the probabilities that one rate is larger than another. All statistical analyses were conducted using NCSS [38] or the R environment [39].

## Results

### Environmental conditions

Salinity at the core reef sites ranged from 31.4 to 36.0, being highest at the end of the dry season (September 2011, October 2012) and lowest during the two wet seasons (Table 1). This pattern is most distinct in the two northern regions (Wet-tropics and Burdekin) that had the highest levels of terrestrial runoff. During most seasons, salinities in the southern-most region (Fitzroy) were slightly higher compared to other regions. The one exception was during the 2013 wet season when major flooding in the Fitzroy River catchment resulted in low (*ca*. 31) and variable salinities. Temperatures were highest during the summer wet season and lowest in June and declined from the north to the south, regardless of season (Table 1).

Measured Chl *a* concentrations varied between 0.11 and 1.3 µg l^−1^. Inorganic nitrogen and phosphorus concentrations were of the order of 0.1 µmol kg^−1^, with elevated NH_4_^+^ and NO_3_^−^/NO_2_^−^ concentrations during the wet season. The highest wet season NO_3_^−^/NO_2_^−^ levels were generally found in the more freshwater influenced geographic regions (Wet-tropics and Fitzroy). In contrast, the highest PO_4_^3−^concentrations were measured during the dry season (Table 1).

### Inorganic carbon dynamics

**Figure 2:**
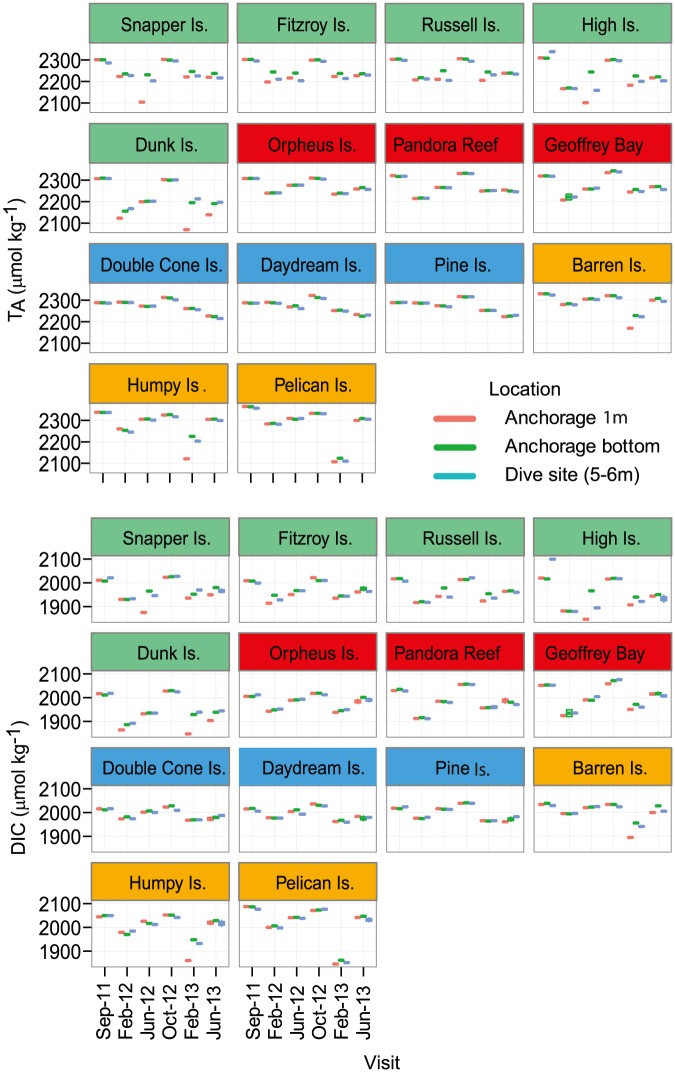
Total Alkalinity (TA) and dissolved inorganic carbon (DIC) data for each island station, sorted from north to south, during each Visit and for each of the three Locations. Regions are colour coded as per Fig. 1. Note that duplicates are plotted but in most cases cannot be distinguished because of the low between-duplicate variance.

With few exceptions, there was no appreciable or consistent difference between data derived from samples collected from the research vessel (near-surface, near-bottom) and by diver on the adjacent coral reef slope (diver collected; Fig. 2). Exceptions were observed during the wet seasons, where the surface sample differed from the two near-bottom samples (e.g. Snapper Island February 2012, Dunk Island February 2013). These samples were characterized as having lower salinity than the contemporaneous near-bottom samples, so the most likely cause was either recent rainfall or freshwater runoff affecting these sites. To facilitate interpretation, we restricted further analyses and statistical tests to samples from the reef slope and the surface samples from the research vessel's anchorage and averaged replicate sub-samples.

**Figure 3:**
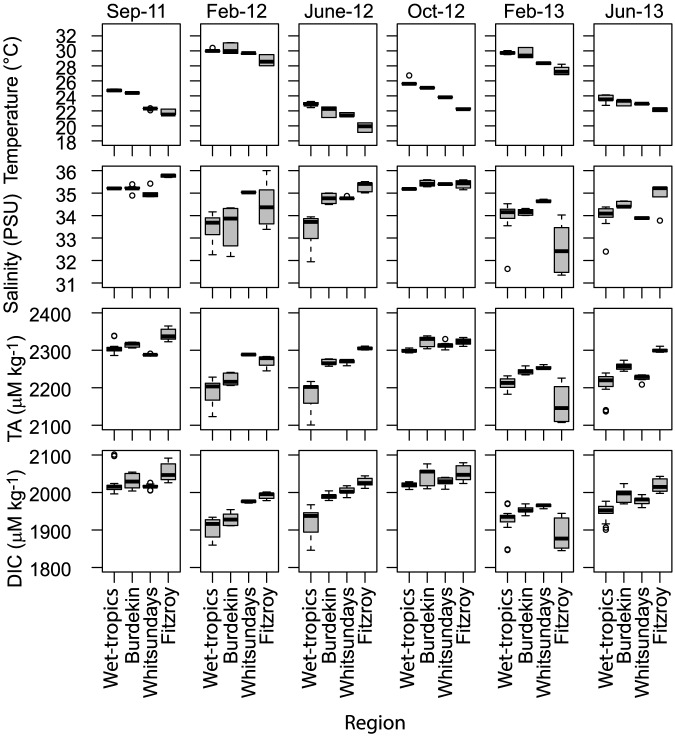
Measured Temperature, Salinity, Total Alkalinity (TA) and dissolved inorganic carbon (DIC) on 14 inshore reefs during six research trips in four geographical regions along the length of the Great Barrier Reef. The box denotes the inter-quartile range, whiskers denote 1.5× the inter-quartile range, the black line indicates the mean, and circles are outliers>1.5× the inter-quartile range.

Total Alkalinity values ranged between 2069 and 2315 µmol kg^−1^. Higher TA levels were generally measured during the late dry season (September–October) and in the regions more influenced by freshwater (Wet-tropics and Fitzroy). There was a general increase from north to south, with the exception of low values in the Fitzroy region in February 2013 (Fig. 2). The ANOVA for inshore TA values showed significant effects of geographic regions and amongst visits (Table 2) with a significant interaction between these factors, indicating that regional trends were dependent on the season sampled (Fig. 3). In general, TA values were closely correlated with salinity (r^2^ = 0.94, p<0.0001; Fig. 2). Thus, salinity normalization (TA_S_) removed a large part of the seasonal variability in TA at the inshore stations; particularly in the Wet-tropics (198 µmol kg^−1^) and Fitzroy (106 µmol kg^−1^) regions where freshwater inputs from rivers were largest (Table 3). In contrast, no salinity related variation was found for the outer shelf reefs (Table 3).

**Table 2:** Mixed model ANOVA for measured parameters Total Alkalinity (TA) and dissolved inorganic carbon (DIC).

			TA			DIC	
	DF	MS	F	p	MS	F	p
**R**egion	3	1.01 10^−03^	22.26	**0.0001**	1.02 10^−03^	21.64	**0.0001**
**I**sland (R)	10	4.54 10^−05^	2.28	0.0179	4.72 10^−05^	2.26	0.0189
**L**ocation	1	2.12 10^−05^	1.06	0.3045	6.38 10^−05^	3.06	0.0830
R x L	3	2.62 10^−05^	1.32	0.2724	2.74 10^−05^	1.32	0.2732
**V**isit	5	1.63 10^−03^	81.85	**<0.0001**	2.19 10^−03^	105.11	**<0.0001**
R x V	15	2.98 10^−04^	14.97	**<0.0001**	2.45 10^−04^	11.77	**<0.0001**
L x V	5	2.22 10^−05^	1.11	0.3568	2.05 10^−05^	0.98	0.4306
R x L x V	15	8.33 10^−06^	0.42	0.9709	8.46 10^−06^	0.41	0.9748
Residual	110	1.99 10^−05^			2.09 10^−05^		

The model tests for differences between four Regions of the Great Barrier Reef (factor “Region”), the vessel's anchorage (0 m) and dive site (“Location”) and six visits over two years of monitoring (“Visit”). “Island” is nested as a random factor in “Region”. Significant (p<0.05) fixed factors are highlighted in bold. All data are log-transformed for analysis. DF: degrees of freedom; MS: mean square; F: F-value for F test.

**Table 3:** A summary of carbon chemistry of water samples collected during 2011 to 2013 in the Great Barrier Reef region.

			TA	TA_S_	DIC	DIC_S_	*p*CO_2_	*p*CO_2, Bio_	*p*CO_2, Temp_
Area	Season	N	µmol kg^− 1^	µmol kg^− 1^	µmol kg^− 1^	µmol kg^− 1^	µatm	µatm	µatm
Wet-tropics	Early dry	20	2196±40	2244±6	1936±32	1978±12	398±24	478±26	380±8
	Late dry	20	2302±10	2262±9	2021±20	1986±19	445±35	490±37	415±12
	Wet	20	2195±40	2239±8	1916±30	1955±11	470±29	401±27	513±5
	All year		2231±60	2249±13	1957±53	1973±20	437±42	437±39	441±54
	Amplitude		269	71	253	123	198	220	150
Burdekin	Early dry	12	2262±9	2256±8	1991±12	1986±19	399±25	489±29	367±12
	Late dry	12	2320±11	2274±9	2038±23	1998±20	442±35	493±37	403±6
	Wet	12	2234±15	2270±31	1941±17	1972±31	458±14	383±15	518±15
	All year		2272±38	2266±20	1990±44	1985±26	433±36	434±44	437±61
	Amplitude		131	100	164	106	134	162	186
Whitsundays	Early dry	12	2248±23	2258±8	1991±16	2000±13	414±26	487±23	367±12
	Late dry	12	2300±14	2261±10	2022±11	1988±13	411±14	467±15	380±13
	Wet	12	2271±18	2252±7	1971±7	1954±5	451±8	377±15	509±15
	All year		2273±28	2257±9	1995±25	1981±22	425±25	427±0	429±59
	Amplitude		107	34	80	68	90	162	157
Fitzroy	Early dry	12	2303±4	2268±27	2023±13	1993±33	376±23	446±22	363±20
	Late dry	12	2332±15	2271±14	2052±21	1998±25	406±31	461±29	379±6
	Wet	12	2214±70	2271±40	1940±62	1990±35	454±39	377±24	496±19
	All year		2283±65	2270±28	2005±61	1994±31	412±45	411±34	416±60
	Amplitude		257	151	242	155	175	145	187
Offshore	Early dry	18	2290±16	2267±19	1969±11	1950±14	368±15	377±22	374±24
	Late dry	23	2308±14	2257±12	1986±12	1943±8	392±27	385±10	389±24
	Wet		n.d	n.d	n.d	n.d	n.d	n.d	n.d
	All year		n.d	n.d	n.d	n.d	n.d	n.d	n.d
	Amplitude		79	86	51	55	103	73	111

The average values (± standard deviation) and amplitude (maximum minus minimum level) for Total Alkalinity (TA), dissolved inorganic carbon (DIC) and salinity normalized TA and DIC (TA_S_, DIC_S_) values are shown, together with partial pressure of carbon dioxide (*p*CO_2_) and effect of biological processes (*p*CO_2,Bio_) and temperature (*p*CO_2, Temp_) on *p*CO_2_ dynamics. N: number of samples used to calculate the average.

**Figure 4:**
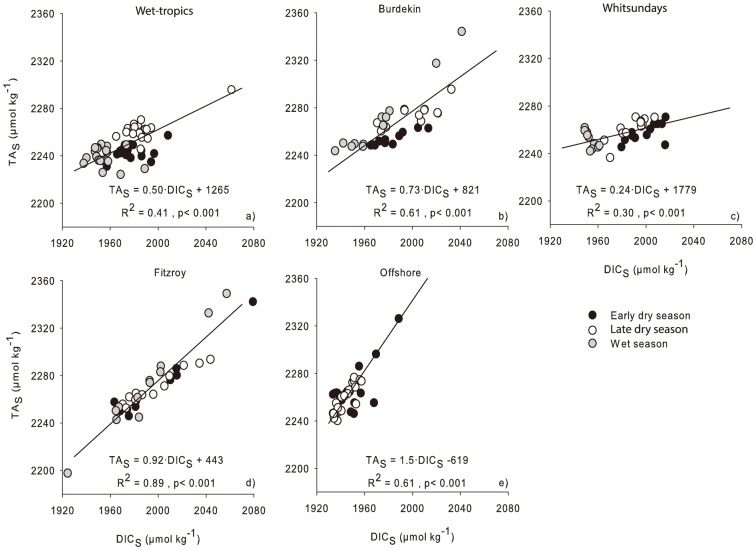
Relationships between salinity normalized dissolved inorganic carbon (DIC_S_) and salinity normalized Total Alkalinity (TA_S_) for the a) Wet-tropics, b) Burdekin, c) Whitsundays, d) Fitzroy and e) offshore shelf regions of the Great Barrier Reef. The regression lines and corresponding equations were obtained using model II linear regression. r^2^: coefficient of determination; p: significance level.

Regional averages of DIC concentrations at inshore reefs ranged between 1930 and 2050 µmol kg^−1^. The within-region variation in DIC concentrations was between 340 and 554 µmol kg^−1^, with the highest concentrations and variation measured in September 2011 and October 2012. Again, there was a distinct north to south DIC gradient during most sampling campaigns (Table 3; Fig. 3). The DIC ANOVA was similar to that for TA, with a strong interaction between Region and Visit (Table 2). As evident in the raw-data plots (Fig. 2), there was no effect of Location (surface water at the anchorage and the reef slope sites) or interactions of this factor with other fixed factors for either TA or DIC (Table 2). Dissolved inorganic carbon was also strongly correlated with salinity (r^2^ = 0.88, p<0.0001) and TA (r^2^ = 0.95, p<0.0001). Salinity normalization of the DIC data (DIC_S_) removed a large part of the seasonal variation, especially in the Wet-tropics (130 µmol kg^−1^) and Fitzroy (87 µmol kg^−1^) region, leaving a residual seasonal variability of 123 and 155 µmol kg^−1^ in those areas (Table 3). The linear relationships between DIC_S_ and TA_S_ had the steepest slopes in the offshore and Fitzroy regions and flattest in the Whitsunday region (Fig. 4). In all cases, the slope value was <2, which resulted in NEP/NEC ratios varying between 7.3 (Whitsundays) and 0.4 (Offshore). This suggests that the importance of calcification in controlling the carbon cycle varies regionally with decreasing importance and larger influence of primary production/respiration in the sequence Offshore (NEP/NEC = 0.4) > Fitzroy (1.2) > Burdekin (1.7) > Wet-tropics (3.0) > Whitsundays (7.3).

**Figure 5:**
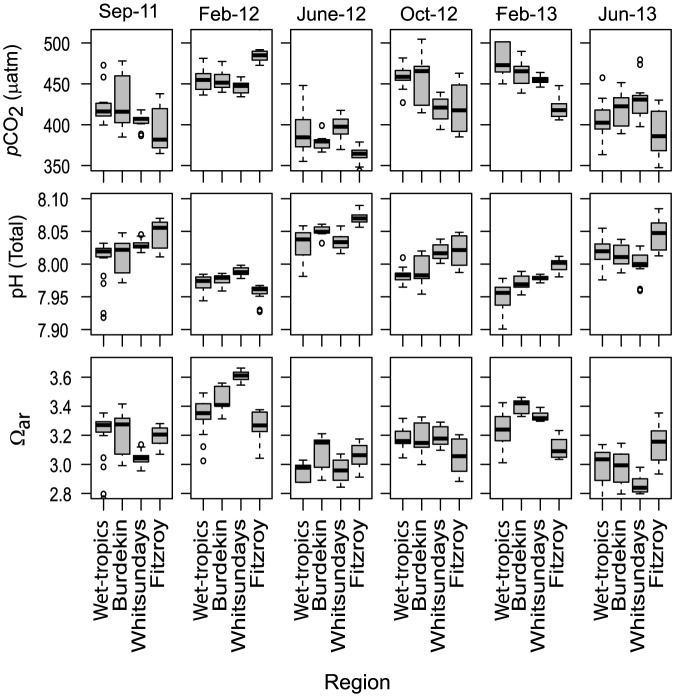
Derived parameters at 14 inshore reefs during six research trips in four geographic regions along the length of the Great Barrier Reef. Ω_ar_ = Aragonite saturation state. The box denotes the inter-quartile range, whiskers denote 1.5× the inter-quartile range, the black line indicates the mean, and circles are outliers >1.5× the inter-quartile range.

Based on the observed patterns of variability for the measured parameters (DIC, TA), the derived parameters *p*CO_2_, pH and Ω_ar_ also exhibited significant interactions between Region and Visit (Table 4). *p*CO_2_ reached concentrations between 340 and 554 µatm, with a decline from north to south during three of the visits (Table 3; Fig. 5). The most distinct temporal pattern in the *p*CO_2_ data (Fig. 5) was an elevation during the wet seasons (total average February 2012: 460 µatm, 1 SD = 19 µatm; February 2013: 460 µatm, 1 SD = 33 µatm), compared to the early dry seasons (June 2012: 383 µatm, 1 SD = 21; June 2013: 410 µatm, 1 SD = 25 µatm) and late dry seasons (September 2011: 416 µatm, 1 SD = 35 µatm; October 2012: 440 µatm, 1 SD = 29 µatm) (Table 3; Fig. 5). Dry season *p*CO_2_ concentrations were slightly elevated relative to present atmospheric values, whereas wet season concentrations were distinctively (∼15%) above atmospheric values. The seasonal fluctuation of *p*CO_2_ concentrations was highest in the Wet-tropics (198 µmol kg^−1^) and Fitzroy (175 µmol kg^−1^) regions (Table 3). Derived pH values varied significantly among trips and a Region x Visit interaction was also significant (Table 4). As expected, seasonal and spatial variations for pH were reversed compared to those of *p*CO_2_ (Fig. 5). Derived pH values were lowest during the wet seasons (February 2012: 7.97, SD = 0.01; February 2013: 7.97, SD = 0.03) and slightly higher in both the early dry seasons (June 2012: 8.04, 1 SD = 0.02; June 2013: 8.02, 1 SD = 0.02) and the late dry seasons (September 2011: 8.02, 1 SD = 0.03, October 2012: 8.00, 1 SD = 0.02). With one exception (February 2012), there was a slight increase in pH from north to south (Fig. 5).

**Table 4:** Mixed model ANOVA for derived parameters partial pressure of carbon dioxide (*p*CO2), pH_[Total]_ and aragonite saturation state (Ω_ar_).

			*p*CO_2_			pH_[total]_			Ω_ar_	
	DF	MS	F	p	MS	F	p	MS	F	p
**R**egion	3	5.53 10^−3^	3.11	0.0757	6.97 10^−3^	6.29	**0.0114**	1.16 10^−3^	1.08	0.4001
**I**sland (R)	10	1.78 10^−3^	4.06	0.0001	1.11 10^−3^	3.7	0.0003	1.07 10^−3^	4.32	<0.0001
**L**ocation	1	2.18 10^−3^	4.97	**0.0278**	1.53 10^−3^	5.1	**0.0259**	4.98 10^−4^	2.02	0.1586
R x L	3	1.60 10^−4^	0.37	0.7784	6.39 10^−5^	0.21	0.8869	2.92 10^−5^	0.12	0.9494
**V**isit	5	0.0255	58.29	**<0.0001**	2.25 10^−2^	75.28	**<0.0001**	1.29 10^−2^	52.09	**<0.0001**
R x V	15	2.26 10^−3^	5.15	**<0.0001**	1.41 10^−3^	4.72	**<0.0001**	1.53 10^−3^	6.19	**<0.0001**
L x V	5	9.84 10^−5^	0.22	0.9512	5.35 10^−5^	0.18	0.9701	5.87 10^−5^	0.24	0.9453
R x L x V	15	1.82 10^−4^	0.41	0.9721	1.21 10^−4^	0.4	0.9753	9.76 10^−5^	0.39	0.9780
Residual	110	4.38 10^−4^			2.99 10^−4^			2.47 10^−4^		

The model tests for differences between four Regions of the Great Barrier Reef (factor “Region”), the vessel's anchorage (0 m) and dive site (“Location”), and six visits over two years of monitoring (“Visit”). “Island” is nested as a random factor in “Region”. Significant (p<0.05) fixed factors are highlighted in bold. With the exception of pH, all data are log-transformed for analysis. DF: degrees of freedom; MS: mean square; F: F-value for F test.

Aragonite saturation state (Ω_ar_) varied between 2.6 and 3.8, with highly significant differences between sampling trips and a significant Region x Visit interaction (Table 4; Fig. 5). Average Ω_ar_ values in the wet seasons (February 2012: 3.39, 1 SD = 0.15; February 2013: 3.25, 1 SD = 0.16) were higher than in the early dry seasons (June 2012: 2.99, 1 SD = 0.13; June 2013: 2.98, 1 SD = 0.16) or in the late dry seasons (September 2011: 3.17, 1 SD = 0.14; October 2012: 3.16, 1 SD = 0.10). Thus, despite higher *p*CO_2_ and lower pH values during the summer wet season, Ω_ar_ were not reduced, but actually increased. This is likely due to higher water temperatures; resulting in lower aragonite solubility in the summer wet season.

In contrast to TA and DIC, the derived parameters *p*CO_2_ and pH exhibited small but significant differences between sampling locations at individual sites (Table 4). The average *p*CO_2_ for reef slope and adjacent open water samples were 432 (1 SD = 42) and 424 (1 SD = 36) µatm, respectively. The overall average pH for the reef slope sites (8.00, 1 SD = 0.04) was slightly lower than the mean for the anchorage sites (8.01, 1 SD = 0.04).

**Figure 6:**
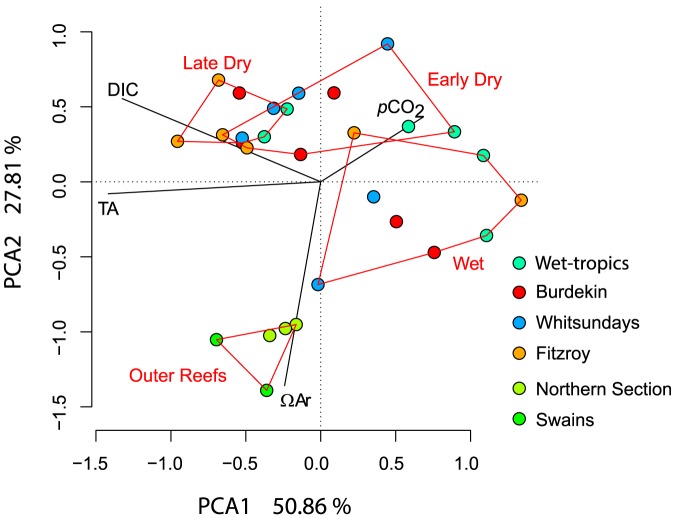
Principal component analysis of carbonate chemistry data for four inshore regions of the Great Barrier Reef (GBR) over three different seasons. Data are contrasted to those from the mid- and outer-shelf reefs of the GBR. Inshore data were pooled over regions per sampling Visit.

Water samples taken at the mid - to outer-shelf reefs over the same study period were less variable than those collected on inshore reefs (Table 3) and the *p*CO_2_ was always closer to atmospheric equilibrium, with resulting higher pH values. The mid - to outer-shelf Ω_ar_ was also clearly higher than most inshore values. A principal component analysis of measured and derived parameters (Fig. 6) separates inshore and mid – to outer-shelf sites, with the main distinguishing factors being Ω_ar_ and *p*CO_2_. In addition, samples taken within the three defined ‘seasons’ (wet, early dry, late dry) clearly group together, with wet season samples distinguished by lower DIC and TA values.

In order to determine whether temperature (T) or biological (B) processes (T/B ratio) primarily controlled *p*CO_2_ dynamics, we used the method proposed by Takahashi et al. [36]. The T/B ratios showed that over an annual cycle, biological effects primarily control *p*CO_2_ dynamics in the Wet-tropics (T/B ratio = 0.7). In the Whitsundays region, temperature and biology contributed equally (1.0), while in the Burdekin (1.2), Fitzroy (1.3) and at the offshore reefs (1.5) the *p*CO_2_ concentrations appear to be primarily controlled by temperature. Thus, for the largest part of the GBR, seasonal *p*CO_2_ changes are most likely controlled by temperature.

## Discussion

Like other coastal areas [40], OA is part of a suite of factors that influence the coral reefs found on the GBR. We analyzed a two year data set collected over a large portion of the GBR to describe seasonal and broad-scale spatial changes in carbon chemistry and contrast those to outer reef areas. Overall, regional variability in carbon system parameters is relatively small. Within the inshore reefs, the largest amount of variation occurred seasonally. In addition, *p*CO_2_ was distinctly higher on inshore compared to offshore reefs.

*p*CO_2_ in the GBR and other coastal waters is influenced by a number of processes, including thermodynamics; air-sea exchange; biological metabolism (photosynthesis, respiration, calcification); and freshwater inputs. Reasons for elevated *p*CO_2_ in the GBR inshore waters especially during the wet season are not fully resolved. In European estuaries, high DIC levels in freshwater can elevate *p*CO_2_ in inshore areas [41]. Although salinity normalization removed a large part of the seasonal amplitude in DIC and TA fluctuations, there is no significant correlation between salinity and *p*CO_2_ in our dataset (r^2^ = 0.02, p = 0.0940). Thus, it is unlikely that freshwater inflow resulted in the elevated *p*CO_2_ in the inshore GBR.

It is also possible that pCO_2_ increase is a consequence of higher calcification (benthic – e.g., corals, foraminifera; or pelagic – e.g., coccolithophorids), resulting from elevated temperatures during the wet seasons [22], [42]. It is difficult to judge the net effect of this on the areas studied, because it would also require more detailed knowledge on accompanying respiration (CO_2_ source) and primary production (CO_2_ sink). The expected slope of the linear regression between DIC_S_ and TA_S_ in systems where calcification is dominating is close to 2.0 e.g., [22], [37]. We found slopes <2 and high (1.2 to 7.3) NEP/NEC ratios for all the inshore regions investigated (Fig. 5), suggesting that processes other than calcification (e.g., photosynthesis, respiration) are largely controlling the carbon cycle on inshore GBR reefs.

Changing *p*CO_2_ outside the equilibrium may also be caused by thermodynamic effects. A seasonal increase at a similar range as observed here (albeit from a lower baseline) was observed near Lady Elliot Island in the southern GBR [20]. Based on the slope of the *p*CO_2_ – temperature relationship, the authors of that study suggested thermodynamic effects as the most likely explanation for elevated *p*CO_2_ values in summer. There was a significant correlation between temperature and *p*CO_2_ in our dataset (r^2^ = 0.53, p<0.0001, *p*CO_2_ = 2.07% [1 SE = 0.1%] * Temp (°C)+5.53), but our value was lower than that found at Lady Elliot Island [3.8 [1 SE = 0.4%], 20], and only about 50% of the expected slope of 4**–**4.2% *p*CO_2_ [43]. However, the regression clearly levels off at >26°C. If data >26°C were excluded, the slope of the regression (r^2^ = 0.42, p <0.0001, *p*CO_2_ = 3.6% [1 SE = 0.4%] * Temp (°C)+5.19) is closer to the theoretical value for temperature controlled systems.

We applied the method proposed by Takahashi et al. [36] to distinguish if seasonal changes in *p*CO_2_ concentrations were controlled by temperature or biological processes. The definition of “biology effect” applied includes biogeochemical processes (e.g., primary production, respiration, calcification), and other processes influencing the CO_2_ such as air-sea exchange and lateral vertical mixing processes [44]. The application of this method suggested a strong temperature control in the southern GBR, as was also proposed by Shaw and McNeil [20]. Northern GBR reefs were impacted more by biological processes. In addition, temporal difference in the controlling factor also existed, with stronger biological and temperature control during wet and dry seasons, respectively (Table 3).

Analysis and modelling of historical trends in coral reef *p*CO_2_ levels worldwide [5] suggests that *p*CO_2_ is sensitive to the P/R ratio of the system and also to overall increases in production and respiration. Sediment and nutrient loads through riverine input into the GBR lagoon have increased several fold since European settlement [45]. Thus, the inshore reefs studied here are characterized by elevated chlorophyll and nutrient values and increased turbidity, especially during the summer months [46]. Higher near-shore turbidity associated with enhanced wet season runoff on the GBR [24] may reduce in situ light availability and thus restrain benthic primary production and favour heterotrophic processes. Although coastal waters in the GBR system are net-autotrophic throughout the year [47], pelagic and benthic heterotrophic processes may also be seasonally enhanced by greater inputs of organic carbon in terrestrial runoff, thus shifting the P/R ratio and increasing the *p*CO_2_ levels in coastal GBR waters.

Dissolved inorganic carbon and TA levels in samples taken during the daytime directly over the coral reef slopes on inshore reefs did not vary greatly from levels taken at the water surface or at ∼9 m depth in nearby open water at the research vessel's anchorages. We consider the small differences in pH and *p*CO_2_ measured as biologically insignificant. This casts some doubt on whether inshore reefs (or at least reef slope areas investigated) on the GBR can take up sufficient DIC during light periods to alter carbon chemistry and buffer increased DIC, as has been suggested for larger offshore reefs [15]–[18]. We assume that currents and wave induced mixing are too high on inshore reefs for the benthos to affect the water carbon chemistry. On reef flats of Lady Elliot Island (southern GBR) *p*CO_2_ can vary between 89 and 1325 µatm and pH between 7.59 and 8.56 depending on time of day and season. pH and *p*CO_2_ in similar ranges were measured on the reef flat of One Tree Island, southern GBR [19] and in lagoonal waters of Heron Island for pH [48], [49]. Diurnal and seasonal changes on a mid-shelf reef flat in the central GBR were also distinct, albeit somewhat less than reported in the latter studies [15]. Compared to that, changes at a back-reef area of another mid-shelf reef were rather small [19]. Thus, it is possible that highly fluctuating values on reef flats reported are extremes and not representative for all habitats, even on mid-shelf reefs. However, further studies including finer scale temporal (i.e., sampling during day and night) and spatial (i.e., comparing different reef habitats) investigations are required to further investigate these dynamics.

Although we did not sample mid- and outer-shelf reefs contemporaneously with the inshore reefs, outer-shelf carbon parameters were less variable than encountered near to the coast. From these data there is clear evidence that inshore reefs at present are subjected to higher *p*CO_2_ and lower Ω_ar_ than on the outer-shelf. Recent measurements at Lady Elliot Island and Davies Reef [15], [18] are in a similar range as observed for mid-shelf reefs here. Both these studies indicated that, even on mid-shelf reefs, night time Ω_ar_ values on reef flats can fall below 3, but all day time values were above the value of the surrounding water, and often above 4. Similarly, carbon chemistry analyses from samples collected in surface waters near mid- and outer-shelf reefs of the GBR showed little spatial or temporal variation (Table 3). *p*CO_2_ values on these reefs were generally at or slightly below equilibrium with the atmosphere, and thus somewhat lower than on inshore reefs in the wet-season (∼20%) and the late dry season (∼10%). Aragonite saturation state on mid- and outer-shelf reefs was in the range of 3.6**–**3.8, also higher than on the inshore reefs studied (∼10**–**20%, depending on the season). The PCA analysis clearly separated data from inshore and mid- and outer-shelf reefs, primarily based on their Ω_ar_ and *p*CO_2_ values.

In general, DIC was lower and less variable on mid- and outer-shelf reefs. As discussed above for seasonal differences, possible sources for CO_2_ are calcification, respiration or input through runoff, but it remains unresolved which factor(s) elevate DIC and *p*CO_2_ inshore. It has been shown from a global dataset that reefs closer to the shore generally have elevated *p*CO_2_ levels [5]. The latter authors suggested that human induced changes in productivity and P/R ratios are the most likely explanation for this. *p*CO_2_ measurements conducted in the GBR lagoon in 1996 [21], [22] showed that all samples had *p*CO_2_ concentrations below atmospheric values. Offshore *p*CO_2_ data collected in the present study were also lower than atmospheric levels, although the difference between atmospheric and near-surface *p*CO_2_ is smaller (Table 5). Kawahata et al. [22] also included data from two inshore stations in the Burdekin and Whitsunday regions, which had *p*CO_2_ close to 325 µatm and Ω_ar_ of ∼3.8. Data collected in the GBR 30 years ago also showed no indication of elevated *p*CO_2_ compared with the atmospheric values (Table 5, Table S2 in File S1) [6], [7]. The rate of increase of *p*CO_2_ on outer reefs measured in this study was on a similar level to that in the atmosphere (1.7 and 1.6 µatm yr ^−1^, respectively, based on dry season data only), which is distinctly below that reported for global reefs based on changes over the last 20 years [5]. The rate of increase in *p*CO_2_ measured on inshore reefs was much closer to the latter estimates. According to the Bayesian modelling there was a 91% and 98% probability that the increase inshore is higher than offshore for the dry and wet seasons, respectively. This comparison suggests that elevated *p*CO_2_ levels on inshore reefs are a relatively recent phenomenon, and it is possible that these are caused by anthropogenically increased sediment and nutrient runoff.

**Table 5:** Summary table of historic (1982/83; 1996) and present (2011 to 2013) data from inshore, mid-and outer-shelf reefs for partial pressure of carbon dioxide in the water (*p*CO_2 water_), aragonite saturation state (Ω_ar_) and partial pressure of carbon dioxide in the atmosphere (*p*CO_2 atmosphere_).

Shelf position	Season	*p*CO_2__water_ (µ atm)	Ω _ar_	*p*CO_2 atmosphere_ ** (µ atm)
**1982/83** *				
Mid-/Outer-shelf reefs	Wet (N = 3)	350 (13)	4.1 (0.2)	Mauna Loa: 343 (2)
	Dry (N = 4)	329 (29)	3.7 (0.9)	
Inshore	Wet (N = 3)	324 (24)	4.0 (0.1)	
	Dry (N = 2)	309 (7)	3.7 (0.3)	
**1996**∧				
All lagoonal samples	Dry (N = 27)	332 (13)	3.8 (0.1)	Cape Ferguson: 361 (1)
Inshore	Dry (N = 2)	325 (1)	3.8 (0.1)	Mauna Loa: 363 (2)
**2011 -13+**				
Mid-/Outer-shelf reefs	Dry (N = 5)	380 (15)	3.6 (0.1)	Cape Ferguson: 394 (1)
				Mauna Loa: 396 (2)
Inshore	Dry (N = 8)	411 (23)	3.1 (0.1)	
	Wet (N = 4)	458 (9)	3.3 (0.2)	
Rate of increase:	*p*CO_2_ (µatm yr^−1^)		Atmospheric:	1.7 (1.6**–**2.0)
			Outer Dry:	1.6 (**−**0.4**–**3.8)
			Inshore Dry:	3.4 (1.8**–**5.0)
			Inshore Wet:	4.5 (2.8**–**6.1)

Standard deviations are given in brackets. Average annual rates of increase of atmospheric *p*CO_2_ and water *p*CO_2_ values are calculated from present and 1982/83 data; ranges are 95% Bayesian confidence intervals.

*1982/83: Barnes et al. unpublished, for raw data see Table S2 in File S1, N = number of reefs surveyed.

∧1996: Kawahata et al. [22].

+2011 - 13: present study, data are averages of the annual regional averages.

**Atmospheric data are given as annual averages from Mauna Loa, Hawaii (available at: http://www.esrl.noaa.gov/gmd/ccgg/trends/#mlo_data) and data for inshore GBR areas at AIMS Townsville (Cape Ferguson: available at: http://ds.data.jma.go.jp/gmd/wdcgg/cgi-bin/wdcgg/accessdata.cgi?index=CFA519S00-CSIRO&select=inventory; only available from 1991 onwards); based on raw data averaged over months (N = 12).

In conclusion, further investigation of the differences between inshore and offshore carbon chemistry and the trophic status of the surrounding waters are crucial for our understanding of the vulnerability of inshore GBR reefs to climate change and OA. However, given the two year dataset presented here and data from offshore reefs it is apparent that, in addition to sporadically enhanced nutrient levels, decreased light and increased sedimentation [24], [25], [50], [51], inshore reefs are subjected to elevated *p*CO_2_ values similar to those expected in the near future under OA scenarios [52]. The present study also confirms that the rate of increase on inshore reefs is faster than for offshore reefs and atmospheric values [5]. Increased *p*CO_2_ can be beneficial for primary producers such as benthic algae, seagrasses and phytoplankton [53], [54] but might decrease coral calcification due to reduced aragonite saturation states. Thus, there is a potential for a further shift from coral reefs to algal-dominated areas. However, it should also be acknowledged that at least to some extent corals on inshore reefs can grow under conditions previously predicted as detrimental for coral reef existence (*p*CO_2_ >450, Ω_ar_<3.3) [8], [55]. To detangle what controls these processes, further water quality and community studies, as well as detailed measurements of calcification and growth of corals and other coral reefs organisms, are needed.

## Supporting Information

File S1 **Table S1**, Raw data of all inshore samples analyzed in the present publications. Station: a unique station code from the Australian Institute of Marine Science (AIMS) database; Island: sample location; Code: a depth related code; Depth (in m): actual sampling depth, 0 m = surface sample, assumed to be on average from 1 m depth; Dupl.: duplicate number; Temp.: temperature (°C); Sal.: salinity; DIC: dissolved inorganic carbon (µmol kg^−1^); TA: Total Alkalinity (µmol kg^−1^); Date: collection date; pH: calculated pH on total scale; *p*CO_2_: calculated partial pressure of CO_2_ (µatm); Ω_Ar_: aragonite saturation state; Time: time of sample collection. **Table S2,** Historic water chemistry data from inshore (Bowling Green Bay, Pandora Reef), mid-shelf (Rib Reef, Davies Reef) and outer-shelf (Myrmidon Reef) reefs of the Great Barrier Reef. Samples are the first sample from the cross-reef transects, and are thus close to the windward reef edge and presumed under little influence of reef metabolism. Samples in Cape Bowling Green are not associated with reefs, but represent inshore water close to the coastline. Methods are the same as described in [6], [7].
(DOCX)
