# Supplementary material for: Coral Reefs on the Edge? Carbon Chemistry on Inshore Reefs of the Great Barrier Reef
Source: PLoS One. 2014 Oct 8;9(10):e109092. doi: 10.1371/journal.pone.0109092 (PMC4190081; doi:10.1371/journal.pone.0109092)
Supplement: File S1 — Table S1, Raw data of all inshore samples analyzed in the present publications. Station: a unique station code from the Australian Institute of Marine Science (AIMS) database; Island: sample location; Code: a depth related code; Depth (in m): actual sampling depth, 0 m = surface sample, assumed to be on average from 1 m depth; Dupl.: duplicate number; Temp.: temperature (°C); Sal.: salinity; DIC: dissolved inorganic carbon (µmol kg−1); TA: Total Alkalinity (µmol kg−1); Date: collection date; pH: calculated pH on total scale; pCO2: calculated partial pressure of CO2 (µatm); ΩAr: aragonite saturation state; Time: time of sample collection. Table S2, Historic water chemistry data from inshore (Bowling Green Bay, Pandora Reef), mid-shelf (Rib Reef, Davies Reef) and outer-shelf (Myrmidon Reef) reefs of the Great Barrier Reef. Samples are the first sample from the cross-reef transects, and are thus close to the windward reef edge and presumed under little influence of reef metabolism. Samples in Cape Bowling Green are not associated with reefs, but represent inshore water close to the coastline. Methods are the same as described in [6], [7]. (DOCX) [file pone.0109092.s001.docx]

Supplementary Table 1. Raw data of all inshore samples analysed in the p[resent publications. Station: a unique station code from the AIMS database; Island: sample location, Code: a depth related code; Depth (in m): actual sampling depth, 0m=surface sample, assumed to be on average from 1m depth; Dupl.: duplicate number; Temp.: temperature (°C), Sal.: salinity, DIC: dissolved inorganic carbon (μmol kg^-1^), TA: Total Alkalinity (μmol kg^-1^), Date: collection date; pH: calculated pH on total scale, pCO2: calculates partial pressure of CO2 (μatm), Ω_ar_ : saturation state of aragonite, Time: time of sample collection.

| **Station** | **Island** | **Code** | **Depth** | **Dupl.** | **Temp.** | **Sal.** | **DIC** | **TA** | **DATE** | **S_CL** | **pH** | **pCO2** | Ω**Ar** | **Time** |
| --- | --- | --- | --- | --- | --- | --- | --- | --- | --- | --- | --- | --- | --- | --- |
| WQM537 | Double Cone | D0 | 7.2 | 1 | 22.3 | 34.9 | 2014 | 2286 | 25-Sep-11 | CR | 8.03 | 403 | 3.0 | 8:10:00 |
| WQM537 | Double Cone | D0 | 7.2 | 2 | 22.3 | 34.9 | 2018 | 2286 | 25-Sep-11 | CR | 8.02 | 412 | 3.0 | 8:10:00 |
| WQM538 | Double Cone | D0 | 0 | 1 | 22.3 | 34.8 | 2016 | 2288 | 25-Sep-11 | R | 8.03 | 402 | 3.1 | 10:03:00 |
| WQM538 | Double Cone | D0 | 0 | 2 | 22.3 | 34.8 | 2015 | 2288 | 25-Sep-11 | R | 8.03 | 401 | 3.1 | 10:03:00 |
| WQM538 | Double Cone | D2 | 26 | 1 | 21.9 | 34.9 | 2012 | 2287 | 25-Sep-11 | R | 8.04 | 390 | 3.1 | 10:03:00 |
| WQM538 | Double Cone | D2 | 26 | 2 | 21.9 | 34.9 | 2012 | 2288 | 25-Sep-11 | R | 8.04 | 388 | 3.1 | 10:03:00 |
| WQM539 | Barren | D0 | 9.5 | 1 | 21.5 | 35.7 | 2026 | 2323 | 26-Sep-11 | CR | 8.07 | 365 | 3.3 | 11:05:00 |
| WQM539 | Barren | D0 | 9.5 | 2 | 21.5 | 35.7 | 2033 | 2326 | 26-Sep-11 | CR | 8.06 | 372 | 3.2 | 11:05:00 |
| WQM540 | Barren | D0 | 0 | 1 | 21.5 | 35.7 | 2035 | 2329 | 26-Sep-11 | R | 8.06 | 371 | 3.2 | 12:30:00 |
| WQM540 | Barren | D0 | 0 | 2 | 21.5 | 35.7 | 2034 | 2331 | 26-Sep-11 | R | 8.07 | 367 | 3.3 | 12:30:00 |
| WQM540 | Barren | D1 | 13 | 1 | 21.2 | 35.7 | 2040 | 2330 | 26-Sep-11 | R | 8.06 | 374 | 3.2 | 12:30:00 |
| WQM540 | Barren | D1 | 13 | 2 | 21.2 | 35.7 | 2038 | 2329 | 26-Sep-11 | R | 8.06 | 372 | 3.2 | 12:30:00 |
| WQM542 | Humpy | D0 | 5.8 | 1 | 21.5 | 35.8 | 2051 | 2337 | 26-Sep-11 | CR | 8.05 | 389 | 3.2 | 14:45:00 |
| WQM542 | Humpy | D0 | 5.8 | 2 | 21.5 | 35.8 | 2048 | 2336 | 26-Sep-11 | CR | 8.05 | 387 | 3.2 | 14:45:00 |
| WQM543 | Humpy | D0 | 0 | 1 | 21.5 | 35.8 | 2044 | 2338 | 26-Sep-11 | R | 8.06 | 375 | 3.3 | 15:57:00 |
| WQM543 | Humpy | D0 | 0 | 2 | 21.5 | 35.8 | 2045 | 2338 | 26-Sep-11 | R | 8.06 | 377 | 3.2 | 15:57:00 |
| WQM543 | Humpy | D1 | 15 | 1 | 21.5 | 35.8 | 2050 | 2336 | 26-Sep-11 | R | 8.05 | 388 | 3.2 | 15:57:00 |
| WQM543 | Humpy | D1 | 15 | 2 | 21.5 | 35.8 | 2050 | 2336 | 26-Sep-11 | R | 8.05 | 390 | 3.2 | 15:57:00 |
| WQM544 | Pelican | D0 | 9 | 1 | 22.2 | 35.8 | 2077 | 2355 | 27-Sep-11 | CR | 8.02 | 424 | 3.1 | 8:20:00 |
| WQM544 | Pelican | D0 | 9 | 2 | 22.2 | 35.8 | 2075 | 2357 | 27-Sep-11 | CR | 8.03 | 417 | 3.1 | 8:20:00 |
| WQM545 | Pelican | D0 | 0 | 1 | 22.2 | 35.8 | 2092 | 2365 | 27-Sep-11 | R | 8.01 | 438 | 3.1 | 11:24:00 |
| WQM545 | Pelican | D0 | 0 | 2 | 22.2 | 35.8 | 2083 | 2364 | 27-Sep-11 | R | 8.02 | 422 | 3.1 | 11:24:00 |
| WQM545 | Pelican | D1 | 8 | 1 | 22.1 | 35.8 | 2085 | 2364 | 27-Sep-11 | R | 8.02 | 423 | 3.1 | 11:24:00 |
| WQM545 | Pelican | D1 | 8 | 2 | 22.1 | 35.8 | 2088 | 2362 | 27-Sep-11 | R | 8.01 | 432 | 3.1 | 11:24:00 |
| WQM546 | Pine | D0 | 8 | 1 | 22.1 | 34.8 | 2026 | 2289 | 28-Sep-11 | CR | 8.02 | 418 | 3.0 | 10:35:00 |
| WQM546 | Pine | D0 | 8 | 2 | 22.1 | 34.8 | 2023 | 2291 | 28-Sep-11 | CR | 8.03 | 408 | 3.0 | 10:35:00 |
| WQM547 | Pine | D0 | 0 | 1 | 22.3 | 34.9 | 2018 | 2289 | 28-Sep-11 | R | 8.03 | 408 | 3.0 | 11:48:00 |
| WQM547 | Pine | D0 | 0 | 2 | 22.3 | 34.9 | 2018 | 2288 | 28-Sep-11 | R | 8.02 | 410 | 3.0 | 11:48:00 |
| WQM547 | Pine | D2 | 24 | 1 | 22.3 | 34.9 | 2015 | 2289 | 28-Sep-11 | R | 8.03 | 402 | 3.1 | 11:48:00 |
| WQM547 | Pine | D2 | 24 | 2 | 22.3 | 34.9 | 2018 | 2289 | 28-Sep-11 | R | 8.03 | 406 | 3.0 | 11:48:00 |
| WQM548 | Daydream | D0 | 5.9 | 1 | 22.3 | 35.0 | 2006 | 2288 | 28-Sep-11 | CR | 8.05 | 386 | 3.1 | 16:40:00 |
| WQM548 | Daydream | D0 | 5.9 | 2 | 22.3 | 35.0 | 2005 | 2285 | 28-Sep-11 | CR | 8.04 | 389 | 3.1 | 16:40:00 |
| WQM549 | Daydream | D0 | 0 | 1 | 22.3 | 35.4 | 2015 | 2288 | 28-Sep-11 | R | 8.03 | 407 | 3.0 | 14:30:00 |
| WQM549 | Daydream | D0 | 0 | 2 | 22.3 | 35.4 | 2015 | 2288 | 28-Sep-11 | R | 8.03 | 408 | 3.0 | 14:30:00 |
| WQM549 | Daydream | D2 | 24 | 1 | 22.3 | 35.0 | 2019 | 2286 | 28-Sep-11 | R | 8.02 | 414 | 3.0 | 14:30:00 |
| WQM549 | Daydream | D2 | 24 | 2 | 22.3 | 35.0 | 2016 | 2287 | 28-Sep-11 | R | 8.03 | 405 | 3.0 | 14:30:00 |
| WQM551 | Geoffrey Bay | D0 | 6 | 1 | 24.4 | 34.9 | 2052 | 2318 | 30-Sep-11 | CR | 7.98 | 465 | 3.0 | 8:05:00 |
| WQM551 | Geoffrey Bay | D0 | 6 | 2 | 24.4 | 34.9 | 2052 | 2318 | 30-Sep-11 | CR | 7.98 | 464 | 3.0 | 8:05:00 |
| WQM552 | Geoffrey Bay | D0 | 0 | 1 | 24.4 | 35.4 | 2054 | 2317 | 30-Sep-11 | R | 7.97 | 478 | 3.0 | 9:25:00 |
| WQM552 | Geoffrey Bay | D0 | 0 | 2 | 24.4 | 35.4 | 2049 | 2322 | 30-Sep-11 | R | 7.99 | 456 | 3.1 | 9:25:00 |
| WQM552 | Geoffrey Bay | D1 | 9 | 1 | 24.4 | 35.4 | 2054 | 2318 | 30-Sep-11 | R | 7.97 | 474 | 3.0 | 9:25:00 |
| WQM552 | Geoffrey Bay | D1 | 9 | 2 | 24.4 | 35.4 | 2053 | 2322 | 30-Sep-11 | R | 7.98 | 465 | 3.1 | 9:25:00 |
| WQM553 | Pandora Reef | D0 | 7.6 | 1 | 24.4 | 35.2 | 2028 | 2318 | 30-Sep-11 | CR | 8.02 | 416 | 3.3 | 14:25:00 |
| WQM553 | Pandora Reef | D0 | 7.6 | 2 | 24.4 | 35.2 | 2029 | 2319 | 30-Sep-11 | CR | 8.02 | 416 | 3.3 | 14:25:00 |
| WQM554 | Pandora Reef | D0 | 0 | 1 | 24.4 | 35.2 | 2030 | 2322 | 30-Sep-11 | R | 8.02 | 414 | 3.3 | 15:25:00 |
| WQM554 | Pandora Reef | D0 | 0 | 2 | 24.4 | 35.2 | 2030 | 2320 | 30-Sep-11 | R | 8.02 | 416 | 3.3 | 15:25:00 |
| WQM554 | Pandora Reef | D1 | 12 | 1 | 24.2 | 35.2 | 2034 | 2320 | 30-Sep-11 | R | 8.02 | 420 | 3.2 | 15:25:00 |
| WQM554 | Pandora Reef | D1 | 12 | 2 | 24.2 | 35.2 | 2036 | 2313 | 30-Sep-11 | R | 8.00 | 437 | 3.1 | 15:25:00 |
| WQM555 | Orpheus | D0 | 4.6 | 1 | 24.4 | 35.3 | 2012 | 2306 | 01-Oct-11 | CR | 8.03 | 404 | 3.3 | 8:20:00 |
| WQM555 | Orpheus | D0 | 4.6 | 2 | 24.4 | 35.2 | 2013 | 2308 | 01-Oct-11 | CR | 8.03 | 401 | 3.3 | 8:20:00 |
| WQM556 | Orpheus | D0 | 0 | 1 | 24.4 | 35.2 | 2004 | 2308 | 01-Oct-11 | R | 8.05 | 385 | 3.4 | 9:51:00 |
| WQM556 | Orpheus | D0 | 0 | 2 | 24.4 | 35.2 | 2006 | 2307 | 01-Oct-11 | R | 8.04 | 392 | 3.4 | 9:51:00 |
| WQM556 | Orpheus | D2 | 28 | 1 | 24.3 | 35.3 | 2005 | 2308 | 01-Oct-11 | R | 8.04 | 388 | 3.4 | 9:51:00 |
| WQM556 | Orpheus | D2 | 28 | 2 | 24.3 | 35.3 | 2005 | 2308 | 01-Oct-11 | R | 8.05 | 386 | 3.4 | 9:51:00 |
| WQM564 | Snapper | D0 | 6.5 | 1 | 24.8 | 35.2 | 2024 | 2286 | 03-Oct-11 | CR | 7.97 | 473 | 3.0 | 8:00:00 |
| WQM564 | Snapper | D0 | 6.5 | 2 | 24.8 | 35.2 | 2018 | 2286 | 03-Oct-11 | CR | 7.98 | 458 | 3.0 | 8:00:00 |
| WQM565 | Snapper | D0 | 0 | 1 | 24.8 | 35.2 | 2014 | 2303 | 03-Oct-11 | R | 8.02 | 420 | 3.3 | 8:45:00 |
| WQM565 | Snapper | D0 | 0 | 2 | 24.8 | 35.2 | 2008 | 2300 | 03-Oct-11 | R | 8.02 | 413 | 3.3 | 8:45:00 |
| WQM565 | Snapper | D1 | 8 | 1 | 24.7 | 35.2 | 2007 | 2301 | 03-Oct-11 | R | 8.03 | 407 | 3.3 | 8:45:00 |
| WQM565 | Snapper | D1 | 8 | 2 | 24.7 | 35.2 | 2008 | 2301 | 03-Oct-11 | R | 8.02 | 410 | 3.3 | 8:45:00 |
| WQM566 | Fitzroy | D0 | 6 | 1 | 24.9 | 35.2 | 2002 | 2297 | 03-Oct-11 | CR | 8.03 | 406 | 3.3 | 14:20:00 |
| WQM566 | Fitzroy | D0 | 6 | 2 | 24.9 | 35.2 | 1996 | 2294 | 03-Oct-11 | CR | 8.03 | 400 | 3.4 | 14:20:00 |
| WQM567 | Fitzroy | D0 | 0 | 1 | 24.9 | 35.2 | 2010 | 2301 | 03-Oct-11 | R | 8.02 | 416 | 3.3 | 15:25:00 |
| WQM567 | Fitzroy | D0 | 0 | 2 | 24.9 | 35.2 | 2008 | 2304 | 03-Oct-11 | R | 8.03 | 406 | 3.3 | 15:25:00 |
| WQM567 | Fitzroy | D1 | 15 | 1 | 24.7 | 35.2 | 2010 | 2304 | 03-Oct-11 | R | 8.03 | 408 | 3.3 | 15:25:00 |
| WQM567 | Fitzroy | D1 | 15 | 2 | 24.7 | 35.2 | 2004 | 2301 | 03-Oct-11 | R | 8.03 | 400 | 3.3 | 15:25:00 |
| WQM569 | High | D0 | 4.5 | 1 | 24.7 | 35.2 | 2101 | 2339 | 04-Oct-11 | CR | 7.92 | 559 | 2.8 | 8:05:00 |
| WQM569 | High | D0 | 4.5 | 2 | 24.7 | 35.2 | 2097 | 2338 | 04-Oct-11 | CR | 7.92 | 549 | 2.8 | 8:05:00 |
| WQM570 | High | D0 | 0 | 1 | 24.7 | 35.2 | 2016 | 2309 | 04-Oct-11 | R | 8.02 | 412 | 3.3 | 10:14:00 |
| WQM570 | High | D0 | 0 | 2 | 24.7 | 35.2 | 2024 | 2310 | 04-Oct-11 | R | 8.01 | 425 | 3.2 | 10:14:00 |
| WQM570 | High | D2 | 20 | 1 | 24.6 | 35.2 | 2016 | 2309 | 04-Oct-11 | R | 8.02 | 410 | 3.3 | 10:14:00 |
| WQM570 | High | D2 | 20 | 2 | 24.6 | 35.2 | 2017 | 2308 | 04-Oct-11 | R | 8.02 | 411 | 3.3 | 10:14:00 |
| WQM571 | Russell | D0 | 6.2 | 1 | 24.6 | 35.2 | 2007 | 2298 | 04-Oct-11 | CR | 8.02 | 409 | 3.3 | 13:25:00 |
| WQM571 | Russell | D0 | 6.2 | 2 | 24.6 | 35.2 | 2007 | 2299 | 04-Oct-11 | CR | 8.03 | 407 | 3.3 | 13:25:00 |
| WQM572 | Russell | D0 | 0 | 1 | 24.6 | 35.2 | 2015 | 2306 | 04-Oct-11 | R | 8.02 | 413 | 3.3 | 11:25:00 |
| WQM572 | Russell | D0 | 0 | 2 | 24.6 | 35.2 | 2018 | 2301 | 04-Oct-11 | R | 8.01 | 427 | 3.2 | 11:25:00 |
| WQM572 | Russell | D2 | 20 | 1 | 24.3 | 35.2 | 2015 | 2305 | 04-Oct-11 | R | 8.02 | 411 | 3.3 | 11:25:00 |
| WQM572 | Russell | D2 | 20 | 2 | 24.3 | 35.2 | 2020 | 2303 | 04-Oct-11 | R | 8.01 | 423 | 3.2 | 11:25:00 |
| WQM574 | Dunk | D0 | 4.5 | 1 | 24.6 | 35.3 | 2018 | 2307 | 05-Oct-11 | CR | 8.02 | 416 | 3.3 | 10:10:00 |
| WQM574 | Dunk | D0 | 4.5 | 2 | 24.6 | 35.3 | 2018 | 2308 | 05-Oct-11 | CR | 8.02 | 416 | 3.3 | 10:10:00 |
| WQM575 | Dunk | D0 | 0 | 1 | 24.6 | 35.3 | 2020 | 2307 | 05-Oct-11 | R | 8.02 | 421 | 3.2 | 11:11:00 |
| WQM575 | Dunk | D0 | 0 | 2 | 24.6 | 35.3 | 2014 | 2306 | 05-Oct-11 | R | 8.02 | 412 | 3.3 | 11:11:00 |
| WQM575 | Dunk | D1 | 5 | 1 | 24.5 | 35.3 | 2013 | 2311 | 05-Oct-11 | R | 8.03 | 401 | 3.3 | 11:11:00 |
| WQM575 | Dunk | D1 | 5 | 2 | 24.5 | 35.3 | 2009 | 2308 | 05-Oct-11 | R | 8.04 | 398 | 3.4 | 11:11:00 |
| WQM583 | Double Cone | D0 | 6.1 | 1 | 29.6 | 35.0 | 1972 | 2289 | 10-Feb-12 | CR | 8.00 | 434 | 3.7 | 8:45:00 |
| WQM583 | Double Cone | D0 | 6.1 | 2 | 29.6 | 35.0 | 1976 | 2289 | 10-Feb-12 | CR | 7.99 | 443 | 3.6 | 8:45:00 |
| WQM584 | Double Cone | D0 | 0 | 1 | 29.6 | 35.0 | 1973 | 2291 | 10-Feb-12 | R | 8.00 | 434 | 3.7 | 10:17:00 |
| WQM584 | Double Cone | D0 | 0 | 2 | 29.6 | 35.0 | 1974 | 2291 | 10-Feb-12 | R | 8.00 | 435 | 3.7 | 10:17:00 |
| WQM584 | Double Cone | D2 | 24 | 1 | 29.5 | 35.0 | 1983 | 2289 | 10-Feb-12 | R | 7.98 | 456 | 3.5 | 10:17:00 |
| WQM584 | Double Cone | D2 | 24 | 2 | 29.5 | 35.0 | 1981 | 2290 | 10-Feb-12 | R | 7.98 | 451 | 3.6 | 10:17:00 |
| WQM586 | Pelican | D0 | 7.4 | 1 | 29.5 | 33.6 | 1998 | 2282 | 11-Feb-12 | CR | 7.96 | 487 | 3.4 | 13:40:00 |
| WQM586 | Pelican | D0 | 7.4 | 2 | 29.5 | 33.6 | 1998 | 2282 | 11-Feb-12 | CR | 7.96 | 486 | 3.4 | 13:40:00 |
| WQM587 | Pelican | D0 | 0 | 1 | 29.5 | 33.4 | 2001 | 2284 | 11-Feb-12 | R | 7.96 | 488 | 3.4 | 14:52:00 |
| WQM587 | Pelican | D0 | 0 | 2 | 29.5 | 33.4 | 1999 | 2283 | 11-Feb-12 | R | 7.96 | 484 | 3.4 | 14:52:00 |
| WQM587 | Pelican | D1 | 7 | 1 | 28.7 | 33.8 | 2007 | 2286 | 11-Feb-12 | R | 7.96 | 488 | 3.3 | 14:52:00 |
| WQM587 | Pelican | D1 | 7 | 2 | 28.7 | 33.8 | 2006 | 2285 | 11-Feb-12 | R | 7.96 | 487 | 3.3 | 14:52:00 |
| WQM588 | Humpy | D0 | 6 | 1 | 28.6 | 34.5 | 1984 | 2245 | 12-Feb-12 | CR | 7.93 | 519 | 3.1 | 8:15:00 |
| WQM588 | Humpy | D0 | 6 | 2 | 28.6 | 34.5 | 1985 | 2245 | 12-Feb-12 | CR | 7.93 | 523 | 3.0 | 8:15:00 |
| WQM589 | Humpy | D0 | 0 | 1 | 28.6 | 34.2 | 1978 | 2260 | 12-Feb-12 | R | 7.97 | 473 | 3.3 | 9:22:00 |
| WQM589 | Humpy | D0 | 0 | 2 | 28.6 | 34.2 | 1980 | 2260 | 12-Feb-12 | R | 7.97 | 476 | 3.3 | 9:22:00 |
| WQM589 | Humpy | D1 | 12 | 1 | 28.3 | 34.6 | 1968 | 2253 | 12-Feb-12 | R | 7.97 | 460 | 3.3 | 9:22:00 |
| WQM589 | Humpy | D1 | 12 | 2 | 28.3 | 34.6 | 1972 | 2253 | 12-Feb-12 | R | 7.97 | 471 | 3.2 | 9:22:00 |
| WQM590 | Barren | D0 | 10.5 | 1 | 28.0 | 35.1 | 1995 | 2279 | 12-Feb-12 | CR | 7.96 | 477 | 3.3 | 11:40:00 |
| WQM590 | Barren | D0 | 10.5 | 2 | 28.0 | 35.1 | 1998 | 2279 | 12-Feb-12 | CR | 7.96 | 483 | 3.2 | 11:40:00 |
| WQM591 | Barren | D0 | 0 | 1 | 28.0 | 36.0 | 1996 | 2278 | 12-Feb-12 | R | 7.95 | 492 | 3.2 | 14:01:00 |
| WQM591 | Barren | D0 | 0 | 2 | 28.0 | 36.0 | 1994 | 2281 | 12-Feb-12 | R | 7.96 | 481 | 3.3 | 14:01:00 |
| WQM591 | Barren | D1 | 15 | 1 | 27.9 | 35.2 | 1994 | 2283 | 12-Feb-12 | R | 7.97 | 465 | 3.3 | 14:01:00 |
| WQM591 | Barren | D1 | 15 | 2 | 27.9 | 35.2 | 1995 | 2284 | 12-Feb-12 | R | 7.97 | 465 | 3.3 | 14:01:00 |
| WQM593 | Pine | D0 | 7.1 | 1 | 29.7 | 35.1 | 1980 | 2287 | 13-Feb-12 | CR | 7.98 | 459 | 3.5 | 11:55:00 |
| WQM594 | Pine | D0 | 0 | 1 | 29.7 | 35.0 | 1974 | 2287 | 13-Feb-12 | R | 7.99 | 445 | 3.6 | 13:27:00 |
| WQM594 | Pine | D0 | 0 | 2 | 29.7 | 35.0 | 1978 | 2287 | 13-Feb-12 | R | 7.98 | 452 | 3.6 | 13:27:00 |
| WQM594 | Pine | D1 | 10 | 1 | 29.7 | 35.0 | 1975 | 2287 | 13-Feb-12 | R | 7.99 | 447 | 3.6 | 13:27:00 |
| WQM594 | Pine | D1 | 10 | 2 | 29.7 | 35.0 | 1975 | 2286 | 13-Feb-12 | R | 7.99 | 448 | 3.6 | 13:27:00 |
| WQM595 | Daydream | D0 | 8 | 1 | 29.7 | 35.0 | 1975 | 2286 | 13-Feb-12 | CR | 7.99 | 449 | 3.6 | 15:30:00 |
| WQM595 | Daydream | D0 | 8 | 2 | 29.7 | 35.0 | 1978 | 2286 | 13-Feb-12 | CR | 7.98 | 456 | 3.6 | 15:30:00 |
| WQM596 | Daydream | D0 | 0 | 1 | 29.7 | 35.1 | 1978 | 2290 | 13-Feb-12 | R | 7.99 | 449 | 3.6 | 16:30:00 |
| WQM596 | Daydream | D0 | 0 | 2 | 29.7 | 35.1 | 1978 | 2290 | 13-Feb-12 | R | 7.99 | 447 | 3.6 | 16:30:00 |
| WQM596 | Daydream | D2 | 22 | 1 | 29.5 | 35.0 | 1976 | 2288 | 13-Feb-12 | R | 7.99 | 443 | 3.6 | 16:30:00 |
| WQM596 | Daydream | D2 | 22 | 2 | 29.5 | 35.0 | 1977 | 2288 | 13-Feb-12 | R | 7.99 | 445 | 3.6 | 16:30:00 |
| WQM598 | Geoffrey Bay | D0 | 0 | 1 | 31.1 | 33.0 | 1922 | 2208 | 15-Feb-12 | R | 7.96 | 472 | 3.4 | 9:28:00 |
| WQM598 | Geoffrey Bay | D0 | 0 | 2 | 31.1 | 33.0 | 1922 | 2208 | 15-Feb-12 | R | 7.96 | 471 | 3.4 | 9:28:00 |
| WQM598 | Geoffrey Bay | D1 | 7 | 1 | 31.1 | 33.0 | 1921 | 2208 | 15-Feb-12 | R | 7.96 | 468 | 3.4 | 9:28:00 |
| WQM598 | Geoffrey Bay | D1 | 7 | 2 | 31.1 | 33.0 | 1921 | 2208 | 15-Feb-12 | R | 7.96 | 468 | 3.4 | 9:28:00 |
| WQM600 | Pandora Reef | D0 | 7.7 | 1 | 31.1 | 34.0 | 1912 | 2216 | 15-Feb-12 | CR | 7.98 | 446 | 3.6 | 14:50:00 |
| WQM600 | Pandora Reef | D0 | 7.7 | 2 | 31.1 | 34.0 | 1911 | 2216 | 15-Feb-12 | CR | 7.98 | 445 | 3.6 | 14:50:00 |
| WQM601 | Pandora Reef | D0 | 0 | 1 | 30.7 | 33.9 | 1912 | 2215 | 15-Feb-12 | R | 7.98 | 440 | 3.5 | 15:55:00 |
| WQM601 | Pandora Reef | D0 | 0 | 2 | 30.7 | 33.9 | 1912 | 2214 | 15-Feb-12 | R | 7.98 | 440 | 3.5 | 15:55:00 |
| WQM601 | Pandora Reef | D1 | 11 | 1 | 30.6 | 33.9 | 1917 | 2216 | 15-Feb-12 | R | 7.98 | 447 | 3.5 | 15:55:00 |
| WQM601 | Pandora Reef | D1 | 11 | 2 | 30.6 | 33.9 | 1915 | 2217 | 15-Feb-12 | R | 7.98 | 440 | 3.5 | 15:55:00 |
| WQM602 | Orpheus | D0 | 6.2 | 1 | 29.7 | 34.3 | 1954 | 2240 | 16-Feb-12 | CR | 7.96 | 477 | 3.3 | 9:00:00 |
| WQM602 | Orpheus | D0 | 6.2 | 2 | 29.7 | 34.3 | 1949 | 2241 | 16-Feb-12 | CR | 7.97 | 461 | 3.4 | 9:00:00 |
| WQM603 | Orpheus | D0 | 0 | 1 | 29.7 | 34.3 | 1943 | 2240 | 16-Feb-12 | R | 7.98 | 452 | 3.4 | 10:12:00 |
| WQM603 | Orpheus | D0 | 0 | 2 | 29.7 | 34.3 | 1942 | 2239 | 16-Feb-12 | R | 7.98 | 451 | 3.4 | 10:12:00 |
| WQM603 | Orpheus | D2 | 22 | 1 | 29.6 | 34.3 | 1950 | 2242 | 16-Feb-12 | R | 7.97 | 462 | 3.4 | 10:12:00 |
| WQM603 | Orpheus | D2 | 22 | 2 | 29.6 | 34.3 | 1947 | 2240 | 16-Feb-12 | R | 7.97 | 459 | 3.4 | 10:12:00 |
| WQM606 | Dunk | D0 | 4.8 | 1 | 30.0 | 33.2 | 1890 | 2168 | 17-Feb-12 | CR | 7.97 | 456 | 3.3 | 12:00:00 |
| WQM606 | Dunk | D0 | 4.8 | 2 | 30.0 | 33.2 | 1894 | 2167 | 17-Feb-12 | CR | 7.96 | 467 | 3.2 | 12:00:00 |
| WQM607 | Dunk | D0 | 0 | 1 | 30.0 | 32.3 | 1868 | 2123 | 17-Feb-12 | R | 7.94 | 481 | 3.0 | 10:09:00 |
| WQM607 | Dunk | D0 | 0 | 2 | 30.0 | 32.3 | 1860 | 2124 | 17-Feb-12 | R | 7.96 | 458 | 3.1 | 10:09:00 |
| WQM607 | Dunk | D1 | 5 | 1 | 30.1 | 32.8 | 1887 | 2155 | 17-Feb-12 | R | 7.96 | 470 | 3.2 | 10:09:00 |
| WQM607 | Dunk | D1 | 5 | 2 | 30.1 | 32.8 | 1886 | 2156 | 17-Feb-12 | R | 7.96 | 465 | 3.2 | 10:09:00 |
| WQM609 | Russell | D0 | 7.8 | 1 | 29.9 | 33.9 | 1918 | 2213 | 18-Feb-12 | CR | 7.98 | 443 | 3.4 | 8:40:00 |
| WQM609 | Russell | D0 | 7.8 | 2 | 29.9 | 33.9 | 1917 | 2211 | 18-Feb-12 | CR | 7.98 | 442 | 3.4 | 8:40:00 |
| WQM610 | Russell | D0 | 0 | 1 | 29.9 | 33.8 | 1917 | 2207 | 18-Feb-12 | R | 7.98 | 447 | 3.4 | 10:18:00 |
| WQM610 | Russell | D0 | 0 | 2 | 29.9 | 33.8 | 1916 | 2208 | 18-Feb-12 | R | 7.98 | 444 | 3.4 | 10:18:00 |
| WQM610 | Russell | D3 | 20 | 1 | 29.9 | 33.8 | 1919 | 2218 | 18-Feb-12 | R | 7.99 | 434 | 3.5 | 10:18:00 |
| WQM610 | Russell | D3 | 20 | 2 | 29.9 | 33.8 | 1921 | 2217 | 18-Feb-12 | R | 7.99 | 440 | 3.4 | 10:18:00 |
| WQM611 | High | D0 | 4.5 | 1 | 30.4 | 33.2 | 1880 | 2166 | 18-Feb-12 | CR | 7.98 | 443 | 3.4 | 12:50:00 |
| WQM611 | High | D0 | 4.5 | 2 | 30.4 | 33.2 | 1878 | 2167 | 18-Feb-12 | CR | 7.98 | 436 | 3.4 | 12:50:00 |
| WQM612 | High | D0 | 0 | 1 | 30.4 | 33.1 | 1880 | 2167 | 18-Feb-12 | R | 7.98 | 440 | 3.4 | 14:11:00 |
| WQM612 | High | D0 | 0 | 2 | 30.4 | 33.1 | 1882 | 2166 | 18-Feb-12 | R | 7.98 | 446 | 3.3 | 14:11:00 |
| WQM612 | High | D2 | 20 | 1 | 30.3 | 33.1 | 1881 | 2170 | 18-Feb-12 | R | 7.98 | 435 | 3.4 | 14:11:00 |
| WQM612 | High | D2 | 20 | 2 | 30.3 | 33.1 | 1880 | 2170 | 18-Feb-12 | R | 7.99 | 432 | 3.4 | 14:11:00 |
| WQM613 | Fitzroy | D0 | 7.2 | 1 | 29.9 | 33.9 | 1928 | 2211 | 19-Feb-12 | CR | 7.96 | 470 | 3.3 | 8:20:00 |
| WQM613 | Fitzroy | D0 | 7.2 | 2 | 29.9 | 33.9 | 1927 | 2211 | 19-Feb-12 | CR | 7.96 | 467 | 3.3 | 8:20:00 |
| WQM614 | Fitzroy | D0 | 0 | 1 | 29.9 | 33.6 | 1913 | 2196 | 19-Feb-12 | R | 7.97 | 458 | 3.3 | 9:16:00 |
| WQM614 | Fitzroy | D0 | 0 | 2 | 29.9 | 33.6 | 1916 | 2200 | 19-Feb-12 | R | 7.97 | 458 | 3.3 | 9:16:00 |
| WQM614 | Fitzroy | D2 | 15 | 1 | 29.6 | 34.4 | 1946 | 2245 | 19-Feb-12 | R | 7.98 | 450 | 3.5 | 9:16:00 |
| WQM614 | Fitzroy | D2 | 15 | 2 | 29.6 | 34.4 | 1949 | 2245 | 19-Feb-12 | R | 7.98 | 456 | 3.4 | 9:16:00 |
| WQM615 | Snapper | D0 | 6.4 | 1 | 30.0 | 34.2 | 1934 | 2228 | 19-Feb-12 | CR | 7.97 | 455 | 3.4 | 16:10:00 |
| WQM615 | Snapper | D0 | 6.4 | 2 | 30.0 | 34.2 | 1932 | 2227 | 19-Feb-12 | CR | 7.97 | 455 | 3.4 | 16:10:00 |
| WQM616 | Snapper | D0 | 0 | 1 | 30.0 | 34.1 | 1932 | 2220 | 19-Feb-12 | R | 7.96 | 467 | 3.4 | 14:51:00 |
| WQM616 | Snapper | D0 | 0 | 2 | 30.0 | 34.1 | 1928 | 2228 | 19-Feb-12 | R | 7.98 | 442 | 3.5 | 14:51:00 |
| WQM616 | Snapper | D1 | 7 | 1 | 29.8 | 34.3 | 1930 | 2235 | 19-Feb-12 | R | 7.99 | 432 | 3.5 | 14:51:00 |
| WQM616 | Snapper | D1 | 7 | 2 | 30.0 | 34.3 | 1929 | 2236 | 19-Feb-12 | R | 7.99 | 433 | 3.6 | 14:51:00 |
| WQM624 | Geoffrey Bay | D0 | 7 | 1 | 30.0 | 32.7 | 1935 | 2221 | 22-Feb-12 | CR | 7.98 | 451 | 3.4 | 7:00:00 |
| WQM624 | Geoffrey Bay | D0 | 7 | 2 | 30.0 | 32.2 | 1936 | 2222 | 22-Feb-12 | CR | 7.99 | 449 | 3.4 | 7:00:00 |
| WQM625 | Geoffrey Bay | D0 | 0 | 1 | 30.0 | 32.2 | 1929 | 2206 | 22-Feb-12 | R | 7.97 | 461 | 3.3 | 7:17:00 |
| WQM625 | Geoffrey Bay | D0 | 0 | 2 | 30.0 | 32.2 | 1926 | 2207 | 22-Feb-12 | R | 7.98 | 453 | 3.4 | 7:17:00 |
| WQM625 | Geoffrey Bay | D1 | 9 | 1 | 29.9 | 33.0 | 1949 | 2235 | 22-Feb-12 | R | 7.98 | 460 | 3.4 | 7:17:00 |
| WQM625 | Geoffrey Bay | D1 | 9 | 2 | 29.9 | 33.0 | 1950 | 2234 | 22-Feb-12 | R | 7.97 | 464 | 3.4 | 7:17:00 |
| WQM627 | High | D0 | 4 | 1 | 23.1 | 33.0 | 1888 | 2158 | 06-Jun-12 | CR | 8.06 | 357 | 3.0 | 14:40:00 |
| WQM627 | High | D0 | 4 | 2 | 23.1 | 33.0 | 1900 | 2160 | 06-Jun-12 | CR | 8.04 | 377 | 2.9 | 14:40:00 |
| WQM628 | High | D0 | 0 | 1 | 23.1 | 31.9 | 1846 | 2101 | 06-Jun-12 | R | 8.05 | 355 | 2.9 | 16:01:00 |
| WQM628 | High | D0 | 0 | 2 | 23.1 | 31.9 | 1847 | 2102 | 06-Jun-12 | R | 8.05 | 357 | 2.9 | 16:01:00 |
| WQM628 | High | D2 | 18 | 1 | 23.2 | 34.5 | 1967 | 2244 | 06-Jun-12 | R | 8.04 | 388 | 3.1 | 16:01:00 |
| WQM628 | High | D2 | 18 | 2 | 23.2 | 34.5 | 1966 | 2245 | 06-Jun-12 | R | 8.04 | 384 | 3.1 | 16:01:00 |
| WQM636 | Snapper | D0 | 6.6 | 1 | 23.2 | 33.9 | 1944 | 2203 | 08-Jun-12 | CR | 8.02 | 404 | 2.9 | 9:40:00 |
| WQM636 | Snapper | D0 | 6.6 | 2 | 23.2 | 33.9 | 1948 | 2203 | 08-Jun-12 | CR | 8.01 | 413 | 2.9 | 9:40:00 |
| WQM637 | Snapper | D0 | 0 | 1 | 23.2 | 32.0 | 1878 | 2104 | 08-Jun-12 | R | 7.99 | 420 | 2.6 | 10:56:00 |
| WQM637 | Snapper | D0 | 0 | 2 | 23.2 | 32.0 | 1872 | 2104 | 08-Jun-12 | R | 8.00 | 408 | 2.7 | 10:56:00 |
| WQM637 | Snapper | D1 | 8 | 1 | 23.8 | 34.2 | 1968 | 2230 | 08-Jun-12 | R | 8.01 | 422 | 3.0 | 10:56:00 |
| WQM637 | Snapper | D1 | 8 | 2 | 23.8 | 34.2 | 1962 | 2232 | 08-Jun-12 | R | 8.02 | 405 | 3.0 | 10:56:00 |
| WQM638 | Fitzroy | D0 | 5.8 | 1 | 22.8 | 33.9 | 1966 | 2203 | 09-Jun-12 | CR | 7.98 | 448 | 2.7 | 8:10:00 |
| WQM638 | Fitzroy | D0 | 5.8 | 2 | 22.8 | 33.9 | 1967 | 2204 | 09-Jun-12 | CR | 7.98 | 448 | 2.7 | 8:10:00 |
| WQM639 | Fitzroy | D0 | 0 | 1 | 22.8 | 33.9 | 1952 | 2217 | 09-Jun-12 | R | 8.03 | 392 | 3.0 | 9:04:00 |
| WQM639 | Fitzroy | D0 | 0 | 2 | 22.8 | 33.9 | 1949 | 2216 | 09-Jun-12 | R | 8.04 | 386 | 3.0 | 9:04:00 |
| WQM639 | Fitzroy | D2 | 14 | 1 | 23.4 | 34.3 | 1967 | 2239 | 09-Jun-12 | R | 8.03 | 397 | 3.1 | 9:04:00 |
| WQM639 | Fitzroy | D2 | 14 | 2 | 23.4 | 34.3 | 1967 | 2240 | 09-Jun-12 | R | 8.03 | 396 | 3.1 | 9:04:00 |
| WQM640 | Russell | D0 | 6.4 | 1 | 22.8 | 33.8 | 1939 | 2204 | 09-Jun-12 | CR | 8.04 | 385 | 3.0 | 12:20:00 |
| WQM640 | Russell | D0 | 6.4 | 2 | 22.8 | 33.8 | 1940 | 2205 | 09-Jun-12 | CR | 8.04 | 385 | 3.0 | 12:20:00 |
| WQM641 | Russell | D0 | 0 | 1 | 22.8 | 33.8 | 1943 | 2209 | 09-Jun-12 | R | 8.04 | 384 | 3.0 | 13:58:00 |
| WQM641 | Russell | D0 | 0 | 2 | 22.8 | 33.8 | 1942 | 2211 | 09-Jun-12 | R | 8.04 | 380 | 3.0 | 13:58:00 |
| WQM641 | Russell | D3 | 20 | 1 | 23.3 | 34.6 | 1977 | 2249 | 09-Jun-12 | R | 8.02 | 403 | 3.1 | 13:58:00 |
| WQM641 | Russell | D3 | 20 | 2 | 23.3 | 34.6 | 1979 | 2251 | 09-Jun-12 | R | 8.02 | 405 | 3.0 | 13:58:00 |
| WQM643 | Dunk | D0 | 5 | 1 | 22.4 | 33.7 | 1936 | 2202 | 10-Jun-12 | CR | 8.05 | 375 | 3.0 | 10:00:00 |
| WQM643 | Dunk | D0 | 5 | 2 | 22.4 | 33.7 | 1934 | 2201 | 10-Jun-12 | CR | 8.05 | 373 | 3.0 | 10:00:00 |
| WQM644 | Dunk | D0 | 0 | 1 | 22.4 | 33.6 | 1932 | 2200 | 10-Jun-12 | R | 8.05 | 370 | 3.0 | 10:53:00 |
| WQM644 | Dunk | D0 | 0 | 2 | 22.4 | 33.6 | 1933 | 2199 | 10-Jun-12 | R | 8.05 | 373 | 3.0 | 10:53:00 |
| WQM644 | Dunk | D1 | 5 | 1 | 22.3 | 33.7 | 1936 | 2201 | 10-Jun-12 | R | 8.05 | 374 | 3.0 | 10:53:00 |
| WQM644 | Dunk | D1 | 5 | 2 | 22.3 | 33.7 | 1934 | 2202 | 10-Jun-12 | R | 8.05 | 369 | 3.0 | 10:53:00 |
| WQM648 | Geoffrey Bay | D0 | 5 | 1 | 21.1 | 34.5 | 2004 | 2263 | 11-Jun-12 | CR | 8.03 | 399 | 2.9 | 8:12:00 |
| WQM648 | Geoffrey Bay | D0 | 5 | 2 | 21.1 | 34.5 | 2004 | 2263 | 11-Jun-12 | CR | 8.03 | 399 | 2.9 | 8:12:00 |
| WQM649 | Geoffrey Bay | D0 | 0 | 1 | 21.1 | 34.5 | 1991 | 2259 | 11-Jun-12 | R | 8.05 | 379 | 3.0 | 9:30:00 |
| WQM649 | Geoffrey Bay | D0 | 0 | 2 | 21.1 | 34.5 | 1990 | 2258 | 11-Jun-12 | R | 8.05 | 380 | 3.0 | 9:30:00 |
| WQM649 | Geoffrey Bay | D1 | 8 | 1 | 21.0 | 34.5 | 1986 | 2258 | 11-Jun-12 | R | 8.06 | 370 | 3.0 | 9:30:00 |
| WQM649 | Geoffrey Bay | D1 | 8 | 2 | 21.0 | 34.5 | 1992 | 2259 | 11-Jun-12 | R | 8.05 | 380 | 3.0 | 9:30:00 |
| WQM650 | Barren | D0 | 8 | 1 | 20.4 | 35.5 | 2024 | 2304 | 13-Jun-12 | CR | 8.06 | 371 | 3.1 | 9:10:00 |
| WQM650 | Barren | D0 | 8 | 2 | 20.4 | 35.5 | 2026 | 2302 | 13-Jun-12 | CR | 8.06 | 376 | 3.0 | 9:10:00 |
| WQM651 | Barren | D0 | 0 | 1 | 20.4 | 35.4 | 2020 | 2304 | 13-Jun-12 | R | 8.07 | 364 | 3.1 | 10:47:00 |
| WQM651 | Barren | D0 | 0 | 2 | 20.4 | 35.4 | 2020 | 2306 | 13-Jun-12 | R | 8.07 | 359 | 3.1 | 10:47:00 |
| WQM651 | Barren | D2 | 14 | 1 | 20.2 | 35.4 | 2024 | 2306 | 13-Jun-12 | R | 8.07 | 363 | 3.1 | 10:47:00 |
| WQM651 | Barren | D2 | 14 | 2 | 20.2 | 35.4 | 2021 | 2306 | 13-Jun-12 | R | 8.08 | 358 | 3.1 | 10:47:00 |
| WQM652 | Pelican | D0 | 7.5 | 1 | 19.1 | 35.1 | 2038 | 2308 | 13-Jun-12 | CR | 8.07 | 366 | 3.0 | 14:10:00 |
| WQM652 | Pelican | D0 | 7.5 | 2 | 19.1 | 35.1 | 2038 | 2309 | 13-Jun-12 | CR | 8.07 | 365 | 3.0 | 14:10:00 |
| WQM653 | Pelican | D0 | 0 | 1 | 19.1 | 35.0 | 2044 | 2307 | 13-Jun-12 | R | 8.06 | 379 | 2.9 | 13:04:00 |
| WQM653 | Pelican | D0 | 0 | 2 | 19.1 | 35.0 | 2038 | 2311 | 13-Jun-12 | R | 8.08 | 360 | 3.0 | 13:04:00 |
| WQM653 | Pelican | D1 | 8 | 1 | 18.9 | 35.0 | 2045 | 2301 | 13-Jun-12 | R | 8.05 | 387 | 2.8 | 13:04:00 |
| WQM653 | Pelican | D1 | 8 | 2 | 18.9 | 35.0 | 2040 | 2309 | 13-Jun-12 | R | 8.07 | 364 | 3.0 | 13:04:00 |
| WQM654 | Humpy | D0 | 7.3 | 1 | 19.9 | 35.4 | 2011 | 2301 | 14-Jun-12 | CR | 8.09 | 343 | 3.2 | 8:05:00 |
| WQM654 | Humpy | D0 | 7.3 | 2 | 19.9 | 35.4 | 2014 | 2301 | 14-Jun-12 | CR | 8.08 | 348 | 3.1 | 8:05:00 |
| WQM655 | Humpy | D0 | 0 | 1 | 19.9 | 35.4 | 2027 | 2304 | 14-Jun-12 | R | 8.07 | 367 | 3.1 | 9:29:00 |
| WQM655 | Humpy | D0 | 0 | 2 | 19.9 | 35.4 | 2025 | 2306 | 14-Jun-12 | R | 8.07 | 360 | 3.1 | 9:29:00 |
| WQM655 | Humpy | D2 | 16 | 1 | 19.9 | 35.4 | 2020 | 2307 | 14-Jun-12 | R | 8.08 | 350 | 3.1 | 9:29:00 |
| WQM655 | Humpy | D2 | 16 | 2 | 19.9 | 35.4 | 2014 | 2305 | 14-Jun-12 | R | 8.09 | 342 | 3.2 | 9:29:00 |
| WQM656 | Pine | D0 | 7.2 | 1 | 21.3 | 34.7 | 2018 | 2272 | 15-Jun-12 | CR | 8.02 | 417 | 2.8 | 8:50:00 |
| WQM656 | Pine | D0 | 7.2 | 2 | 21.3 | 34.7 | 2008 | 2267 | 15-Jun-12 | CR | 8.03 | 404 | 2.9 | 8:50:00 |
| WQM657 | Pine | D0 | 0 | 1 | 21.3 | 34.7 | 2016 | 2275 | 15-Jun-12 | R | 8.03 | 406 | 2.9 | 10:03:00 |
| WQM657 | Pine | D0 | 0 | 2 | 21.3 | 34.7 | 2016 | 2274 | 15-Jun-12 | R | 8.02 | 410 | 2.9 | 10:03:00 |
| WQM657 | Pine | D2 | 22 | 1 | 21.3 | 34.7 | 2012 | 2274 | 15-Jun-12 | R | 8.03 | 400 | 2.9 | 10:03:00 |
| WQM657 | Pine | D2 | 22 | 2 | 21.3 | 34.7 | 2016 | 2273 | 15-Jun-12 | R | 8.02 | 411 | 2.9 | 10:03:00 |
| WQM658 | Daydream | D0 | 6.4 | 1 | 21.4 | 34.8 | 1986 | 2263 | 15-Jun-12 | CR | 8.06 | 370 | 3.1 | 11:35:00 |
| WQM658 | Daydream | D0 | 6.4 | 2 | 21.4 | 34.8 | 2000 | 2259 | 15-Jun-12 | CR | 8.03 | 404 | 2.9 | 11:35:00 |
| WQM659 | Daydream | D0 | 0 | 1 | 21.4 | 34.8 | 2000 | 2270 | 15-Jun-12 | R | 8.04 | 385 | 3.0 | 12:52:00 |
| WQM659 | Daydream | D0 | 0 | 2 | 21.4 | 34.8 | 2008 | 2267 | 15-Jun-12 | R | 8.02 | 407 | 2.9 | 12:52:00 |
| WQM659 | Daydream | D2 | 21 | 1 | 21.4 | 34.8 | 2010 | 2274 | 15-Jun-12 | R | 8.03 | 399 | 2.9 | 12:52:00 |
| WQM659 | Daydream | D2 | 21 | 2 | 21.4 | 34.8 | 2013 | 2274 | 15-Jun-12 | R | 8.03 | 405 | 2.9 | 12:52:00 |
| WQM660 | Double Cone | D0 | 5 | 1 | 21.8 | 34.9 | 2001 | 2273 | 15-Jun-12 | CR | 8.04 | 390 | 3.0 | 15:25:00 |
| WQM660 | Double Cone | D0 | 5 | 2 | 21.8 | 34.9 | 2000 | 2272 | 15-Jun-12 | CR | 8.04 | 389 | 3.0 | 15:25:00 |
| WQM661 | Double Cone | D0 | 0 | 1 | 21.8 | 34.8 | 2003 | 2273 | 15-Jun-12 | R | 8.04 | 391 | 3.0 | 14:17:00 |
| WQM661 | Double Cone | D0 | 0 | 2 | 21.8 | 34.8 | 2001 | 2273 | 15-Jun-12 | R | 8.04 | 388 | 3.0 | 14:17:00 |
| WQM661 | Double Cone | D2 | 20 | 1 | 21.8 | 34.9 | 2004 | 2273 | 15-Jun-12 | R | 8.03 | 396 | 3.0 | 14:17:00 |
| WQM661 | Double Cone | D2 | 20 | 2 | 21.8 | 34.9 | 2009 | 2269 | 15-Jun-12 | R | 8.02 | 413 | 2.9 | 14:17:00 |
| WQM663 | Pandora Reef | D0 | 6.4 | 1 | 22.3 | 34.8 | 1981 | 2264 | 17-Jun-12 | CR | 8.06 | 372 | 3.2 | 11:55:00 |
| WQM663 | Pandora Reef | D0 | 6.4 | 2 | 22.3 | 34.8 | 1979 | 2265 | 17-Jun-12 | CR | 8.06 | 367 | 3.2 | 11:55:00 |
| WQM664 | Pandora Reef | D0 | 0 | 1 | 22.3 | 34.8 | 1987 | 2266 | 17-Jun-12 | R | 8.05 | 380 | 3.1 | 10:55:00 |
| WQM664 | Pandora Reef | D0 | 0 | 2 | 22.3 | 34.8 | 1983 | 2267 | 17-Jun-12 | R | 8.06 | 371 | 3.2 | 10:55:00 |
| WQM664 | Pandora Reef | D1 | 10 | 1 | 22.1 | 34.8 | 1981 | 2265 | 17-Jun-12 | R | 8.06 | 370 | 3.1 | 10:55:00 |
| WQM664 | Pandora Reef | D1 | 10 | 2 | 22.1 | 34.8 | 1985 | 2267 | 17-Jun-12 | R | 8.05 | 374 | 3.1 | 10:55:00 |
| WQM665 | Orpheus | D0 | 5 | 1 | 22.5 | 35.0 | 1993 | 2277 | 18-Jun-12 | CR | 8.05 | 380 | 3.2 | 9:20:00 |
| WQM665 | Orpheus | D0 | 5 | 2 | 22.5 | 35.0 | 1994 | 2277 | 18-Jun-12 | CR | 8.05 | 383 | 3.1 | 9:20:00 |
| WQM666 | Orpheus | D0 | 0 | 1 | 22.5 | 35.0 | 1987 | 2276 | 18-Jun-12 | R | 8.06 | 371 | 3.2 | 7:59:00 |
| WQM666 | Orpheus | D0 | 0 | 2 | 22.5 | 35.0 | 1991 | 2276 | 18-Jun-12 | R | 8.05 | 379 | 3.2 | 7:59:00 |
| WQM666 | Orpheus | D2 | 26 | 1 | 22.5 | 35.0 | 1991 | 2277 | 18-Jun-12 | R | 8.05 | 377 | 3.2 | 7:59:00 |
| WQM666 | Orpheus | D2 | 26 | 2 | 22.5 | 35.0 | 1990 | 2276 | 18-Jun-12 | R | 8.05 | 376 | 3.2 | 7:59:00 |
| WQM667 | Humpy | D0 | 8.4 | 1 | 22.2 | 35.4 | 2040 | 2318 | 04-Oct-12 | CR | 8.03 | 407 | 3.1 | 11:30:00 |
| WQM667 | Humpy | D0 | 8.4 | 2 | 22.2 | 35.4 | 2043 | 2315 | 04-Oct-12 | CR | 8.02 | 417 | 3.0 | 11:30:00 |
| WQM668 | Humpy | D0 | 0 | 1 | 22.2 | 35.4 | 2051 | 2325 | 04-Oct-12 | R | 8.02 | 418 | 3.1 | 13:11:00 |
| WQM668 | Humpy | D0 | 0 | 2 | 22.2 | 35.4 | 2053 | 2325 | 04-Oct-12 | R | 8.02 | 421 | 3.1 | 13:11:00 |
| WQM668 | Humpy | D1 | 13 | 1 | 22.1 | 35.5 | 2051 | 2326 | 04-Oct-12 | R | 8.02 | 414 | 3.1 | 13:11:00 |
| WQM668 | Humpy | D1 | 13 | 2 | 22.1 | 35.5 | 2053 | 2327 | 04-Oct-12 | R | 8.02 | 415 | 3.1 | 13:11:00 |
| WQM669 | Barren | D0 | 9.5 | 1 | 22.2 | 35.5 | 2024 | 2313 | 04-Oct-12 | CR | 8.05 | 385 | 3.2 | 14:50:00 |
| WQM669 | Barren | D0 | 9.5 | 2 | 22.2 | 35.5 | 2024 | 2311 | 04-Oct-12 | CR | 8.04 | 389 | 3.2 | 14:50:00 |
| WQM670 | Barren | D0 | 0 | 1 | 22.2 | 35.6 | 2034 | 2322 | 04-Oct-12 | R | 8.05 | 390 | 3.2 | 16:00:00 |
| WQM670 | Barren | D0 | 0 | 2 | 22.2 | 35.6 | 2035 | 2320 | 04-Oct-12 | R | 8.04 | 394 | 3.2 | 16:00:00 |
| WQM670 | Barren | D1 | 13 | 1 | 22.2 | 35.5 | 2035 | 2321 | 04-Oct-12 | R | 8.04 | 392 | 3.2 | 16:00:00 |
| WQM670 | Barren | D1 | 13 | 2 | 22.2 | 35.5 | 2034 | 2321 | 04-Oct-12 | R | 8.05 | 390 | 3.2 | 16:00:00 |
| WQM671 | Pelican | D0 | 6.9 | 1 | 22.4 | 35.1 | 2080 | 2334 | 05-Oct-12 | CR | 7.99 | 463 | 2.9 | 8:30:00 |
| WQM671 | Pelican | D0 | 6.9 | 2 | 22.4 | 35.1 | 2074 | 2328 | 05-Oct-12 | CR | 7.99 | 462 | 2.9 | 8:30:00 |
| WQM672 | Pelican | D0 | 0 | 1 | 22.4 | 35.2 | 2071 | 2332 | 05-Oct-12 | R | 8.00 | 447 | 3.0 | 10:21:00 |
| WQM672 | Pelican | D0 | 0 | 2 | 22.4 | 35.2 | 2072 | 2332 | 05-Oct-12 | R | 8.00 | 450 | 2.9 | 10:21:00 |
| WQM672 | Pelican | D1 | 8 | 1 | 22.1 | 35.2 | 2074 | 2333 | 05-Oct-12 | R | 8.00 | 450 | 2.9 | 10:21:00 |
| WQM672 | Pelican | D1 | 8 | 2 | 22.1 | 35.2 | 2073 | 2332 | 05-Oct-12 | R | 8.00 | 448 | 2.9 | 10:21:00 |
| WQM674 | Pine | D0 | 7.2 | 1 | 23.9 | 35.4 | 2040 | 2314 | 06-Oct-12 | CR | 8.00 | 440 | 3.1 | 10:30:00 |
| WQM674 | Pine | D0 | 7.2 | 2 | 23.9 | 35.4 | 2038 | 2318 | 06-Oct-12 | CR | 8.01 | 429 | 3.2 | 10:30:00 |
| WQM675 | Pine | D0 | 0 | 1 | 23.9 | 35.4 | 2039 | 2317 | 06-Oct-12 | R | 8.01 | 433 | 3.1 | 11:55:00 |
| WQM675 | Pine | D0 | 0 | 2 | 23.9 | 35.4 | 2039 | 2317 | 06-Oct-12 | R | 8.01 | 433 | 3.1 | 11:55:00 |
| WQM675 | Pine | D2 | 22 | 1 | 23.8 | 35.4 | 2040 | 2316 | 06-Oct-12 | R | 8.00 | 435 | 3.1 | 11:55:00 |
| WQM675 | Pine | D2 | 22 | 2 | 23.8 | 35.4 | 2042 | 2315 | 06-Oct-12 | R | 8.00 | 442 | 3.1 | 11:55:00 |
| WQM676 | Daydream | D0 | 8.8 | 1 | 23.8 | 35.4 | 2027 | 2306 | 06-Oct-12 | CR | 8.01 | 426 | 3.1 | 13:50:00 |
| WQM676 | Daydream | D0 | 8.8 | 2 | 23.8 | 35.4 | 2028 | 2311 | 06-Oct-12 | CR | 8.02 | 421 | 3.2 | 13:50:00 |
| WQM677 | Daydream | D0 | 0 | 1 | 23.8 | 35.4 | 2032 | 2315 | 06-Oct-12 | R | 8.02 | 421 | 3.2 | 15:03:00 |
| WQM677 | Daydream | D0 | 0 | 2 | 23.8 | 35.4 | 2040 | 2330 | 06-Oct-12 | R | 8.03 | 413 | 3.3 | 15:03:00 |
| WQM677 | Daydream | D2 | 22 | 1 | 23.6 | 35.4 | 2032 | 2311 | 06-Oct-12 | R | 8.01 | 423 | 3.1 | 15:03:00 |
| WQM677 | Daydream | D2 | 22 | 2 | 23.6 | 35.4 | 2029 | 2313 | 06-Oct-12 | R | 8.02 | 415 | 3.2 | 15:03:00 |
| WQM678 | Double Cone | D0 | 6.2 | 1 | 23.8 | 35.4 | 2009 | 2301 | 07-Oct-12 | CR | 8.03 | 399 | 3.3 | 14:50:00 |
| WQM678 | Double Cone | D0 | 6.2 | 2 | 23.8 | 35.4 | 2009 | 2303 | 07-Oct-12 | CR | 8.04 | 394 | 3.3 | 14:50:00 |
| WQM679 | Double Cone | D0 | 0 | 1 | 23.8 | 35.4 | 2027 | 2314 | 07-Oct-12 | R | 8.02 | 412 | 3.2 | 15:51:00 |
| WQM679 | Double Cone | D0 | 0 | 2 | 23.8 | 35.4 | 2020 | 2312 | 07-Oct-12 | R | 8.03 | 402 | 3.3 | 15:51:00 |
| WQM679 | Double Cone | D2 | 22 | 1 | 23.8 | 35.4 | 2029 | 2311 | 07-Oct-12 | R | 8.02 | 421 | 3.2 | 15:51:00 |
| WQM679 | Double Cone | D2 | 22 | 2 | 23.8 | 35.4 | 2028 | 2310 | 07-Oct-12 | R | 8.02 | 421 | 3.2 | 15:51:00 |
| WQM681 | Geoffrey Bay | D0 | 5.6 | 1 | 25.1 | 35.6 | 2076 | 2338 | 08-Oct-12 | CR | 7.95 | 505 | 3.0 | 8:10:00 |
| WQM681 | Geoffrey Bay | D0 | 5.6 | 2 | 25.1 | 35.6 | 2075 | 2338 | 08-Oct-12 | CR | 7.96 | 501 | 3.0 | 8:10:00 |
| WQM682 | Geoffrey Bay | D0 | 0 | 1 | 25.1 | 35.6 | 2059 | 2333 | 08-Oct-12 | R | 7.98 | 471 | 3.1 | 10:06:00 |
| WQM682 | Geoffrey Bay | D0 | 0 | 2 | 25.1 | 35.6 | 2059 | 2336 | 08-Oct-12 | R | 7.98 | 466 | 3.2 | 10:06:00 |
| WQM682 | Geoffrey Bay | D1 | 8 | 1 | 24.6 | 35.6 | 2072 | 2343 | 08-Oct-12 | R | 7.98 | 475 | 3.1 | 10:06:00 |
| WQM682 | Geoffrey Bay | D1 | 8 | 2 | 24.6 | 35.6 | 2071 | 2343 | 08-Oct-12 | R | 7.98 | 473 | 3.1 | 10:06:00 |
| WQM683 | Pandora Reef | D0 | 6.9 | 1 | 25.0 | 35.4 | 2053 | 2330 | 09-Oct-12 | CR | 7.99 | 461 | 3.2 | 8:10:00 |
| WQM683 | Pandora Reef | D0 | 6.9 | 2 | 25.0 | 35.4 | 2058 | 2330 | 09-Oct-12 | CR | 7.98 | 472 | 3.1 | 8:10:00 |
| WQM684 | Pandora Reef | D0 | 0 | 1 | 25.0 | 35.4 | 2056 | 2330 | 09-Oct-12 | R | 7.98 | 468 | 3.1 | 9:15:00 |
| WQM684 | Pandora Reef | D0 | 0 | 2 | 25.0 | 35.4 | 2055 | 2331 | 09-Oct-12 | R | 7.98 | 465 | 3.1 | 9:15:00 |
| WQM684 | Pandora Reef | D1 | 10 | 1 | 25.0 | 35.4 | 2057 | 2331 | 09-Oct-12 | R | 7.98 | 466 | 3.1 | 9:15:00 |
| WQM684 | Pandora Reef | D1 | 10 | 2 | 25.0 | 35.4 | 2057 | 2332 | 09-Oct-12 | R | 7.98 | 467 | 3.1 | 9:15:00 |
| WQM685 | Orpheus | D0 | 4.5 | 1 | 25.1 | 35.3 | 2010 | 2305 | 09-Oct-12 | CR | 8.02 | 415 | 3.3 | 13:20:00 |
| WQM685 | Orpheus | D0 | 4.5 | 2 | 25.1 | 35.3 | 2014 | 2304 | 09-Oct-12 | CR | 8.01 | 423 | 3.3 | 13:20:00 |
| WQM686 | Orpheus | D0 | 0 | 1 | 25.1 | 35.3 | 2018 | 2308 | 09-Oct-12 | R | 8.01 | 425 | 3.3 | 14:20:00 |
| WQM686 | Orpheus | D0 | 0 | 2 | 25.1 | 35.3 | 2018 | 2312 | 09-Oct-12 | R | 8.02 | 420 | 3.3 | 14:20:00 |
| WQM686 | Orpheus | D2 | 28 | 1 | 25.1 | 35.3 | 2020 | 2308 | 09-Oct-12 | R | 8.01 | 426 | 3.3 | 14:20:00 |
| WQM686 | Orpheus | D2 | 28 | 2 | 25.1 | 35.3 | 2018 | 2307 | 09-Oct-12 | R | 8.01 | 425 | 3.3 | 14:20:00 |
| WQM689 | Dunk | D0 | 5 | 1 | 25.6 | 35.2 | 2023 | 2300 | 10-Oct-12 | CR | 7.98 | 459 | 3.1 | 10:10:00 |
| WQM689 | Dunk | D0 | 5 | 2 | 25.6 | 35.2 | 2025 | 2303 | 10-Oct-12 | CR | 7.98 | 457 | 3.2 | 10:10:00 |
| WQM690 | Dunk | D0 | 0 | 1 | 25.6 | 35.2 | 2029 | 2302 | 10-Oct-12 | R | 7.98 | 466 | 3.1 | 11:10:00 |
| WQM690 | Dunk | D0 | 0 | 2 | 25.6 | 35.2 | 2027 | 2303 | 10-Oct-12 | R | 7.98 | 462 | 3.1 | 11:10:00 |
| WQM690 | Dunk | D1 | 5 | 1 | 25.1 | 35.2 | 2027 | 2301 | 10-Oct-12 | R | 7.99 | 456 | 3.1 | 11:10:00 |
| WQM690 | Dunk | D1 | 5 | 2 | 25.1 | 35.2 | 2032 | 2298 | 10-Oct-12 | R | 7.97 | 471 | 3.0 | 11:10:00 |
| WQM694 | Russell | D0 | 6.5 | 1 | 25.6 | 35.2 | 2021 | 2293 | 11-Oct-12 | CR | 7.98 | 466 | 3.1 | 8:10:00 |
| WQM694 | Russell | D0 | 6.5 | 2 | 25.6 | 35.2 | 2020 | 2296 | 11-Oct-12 | CR | 7.98 | 458 | 3.1 | 8:10:00 |
| WQM695 | Russell | D0 | 0 | 2 | 25.6 | 35.2 | 2014 | 2306 | 11-Oct-12 | R | 8.01 | 427 | 3.3 | 9:05:00 |
| WQM695 | Russell | D3 | 20 | 1 | 25.4 | 35.2 | 2015 | 2304 | 11-Oct-12 | R | 8.01 | 429 | 3.3 | 9:05:00 |
| WQM695 | Russell | D3 | 20 | 2 | 25.4 | 35.2 | 2012 | 2305 | 11-Oct-12 | R | 8.01 | 423 | 3.3 | 9:05:00 |
| WQM696 | High | D0 | 5.5 | 1 | 25.7 | 35.2 | 2019 | 2297 | 11-Oct-12 | CR | 7.99 | 454 | 3.2 | 10:50:00 |
| WQM696 | High | D0 | 5.5 | 2 | 25.7 | 35.2 | 2015 | 2297 | 11-Oct-12 | CR | 7.99 | 447 | 3.2 | 10:50:00 |
| WQM697 | High | D0 | 0 | 1 | 25.7 | 35.2 | 2015 | 2299 | 11-Oct-12 | R | 8.00 | 443 | 3.2 | 11:59:00 |
| WQM697 | High | D2 | 20 | 1 | 25.5 | 35.2 | 2018 | 2303 | 11-Oct-12 | R | 8.00 | 439 | 3.2 | 11:59:00 |
| WQM697 | High | D2 | 20 | 2 | 25.5 | 35.2 | 2019 | 2301 | 11-Oct-12 | R | 7.99 | 445 | 3.2 | 11:59:00 |
| WQM698 | Fitzroy | D0 | 5.6 | 1 | 26.7 | 35.1 | 2011 | 2294 | 11-Oct-12 | CR | 7.98 | 461 | 3.2 | 14:20:00 |
| WQM698 | Fitzroy | D0 | 5.6 | 2 | 26.7 | 35.1 | 2008 | 2294 | 11-Oct-12 | CR | 7.98 | 455 | 3.3 | 14:20:00 |
| WQM699 | Fitzroy | D0 | 0 | 1 | 26.7 | 35.2 | 2022 | 2297 | 11-Oct-12 | R | 7.96 | 482 | 3.2 | 15:31:00 |
| WQM699 | Fitzroy | D0 | 0 | 2 | 26.7 | 35.2 | 2020 | 2302 | 11-Oct-12 | R | 7.98 | 468 | 3.2 | 15:31:00 |
| WQM699 | Fitzroy | D2 | 14 | 1 | 25.3 | 35.2 | 2010 | 2300 | 11-Oct-12 | R | 8.01 | 424 | 3.3 | 15:31:00 |
| WQM699 | Fitzroy | D2 | 14 | 2 | 25.3 | 35.2 | 2009 | 2301 | 11-Oct-12 | R | 8.01 | 420 | 3.3 | 15:31:00 |
| WQM707 | Snapper | D0 | 7.5 | 1 | 25.5 | 35.2 | 2029 | 2295 | 13-Oct-12 | CR | 7.97 | 478 | 3.0 | 8:10:00 |
| WQM707 | Snapper | D0 | 7.5 | 2 | 25.5 | 35.2 | 2025 | 2296 | 13-Oct-12 | CR | 7.97 | 468 | 3.1 | 8:10:00 |
| WQM708 | Snapper | D0 | 0 | 1 | 25.5 | 35.2 | 2025 | 2301 | 13-Oct-12 | R | 7.98 | 457 | 3.2 | 9:16:00 |
| WQM708 | Snapper | D0 | 0 | 2 | 25.5 | 35.2 | 2022 | 2304 | 13-Oct-12 | R | 7.99 | 446 | 3.2 | 9:16:00 |
| WQM708 | Snapper | D1 | 8 | 1 | 25.5 | 35.2 | 2025 | 2300 | 13-Oct-12 | R | 7.98 | 460 | 3.1 | 9:16:00 |
| WQM708 | Snapper | D1 | 8 | 2 | 25.5 | 35.2 | 2026 | 2300 | 13-Oct-12 | R | 7.98 | 462 | 3.1 | 9:16:00 |
| WQM716 | Snapper | D0 | 7.1 | 1 | 29.9 | 34.5 | 1971 | 2226 | 16-Feb-13 | CR | 7.90 | 554 | 3.0 | 7:10:00 |
| WQM716 | Snapper | D0 | 7.1 | 2 | 29.9 | 34.5 | 1969 | 2225 | 16-Feb-13 | CR | 7.90 | 553 | 3.0 | 7:10:00 |
| WQM717 | Snapper | D0 | 0 | 1 | 29.9 | 34.2 | 1936 | 2220 | 16-Feb-13 | R | 7.96 | 474 | 3.3 | 8:05:00 |
| WQM717 | Snapper | D0 | 0 | 2 | 29.9 | 34.2 | 1935 | 2222 | 16-Feb-13 | R | 7.96 | 468 | 3.3 | 8:05:00 |
| WQM717 | Snapper | D1 | 8 | 1 | 29.9 | 34.6 | 1952 | 2247 | 16-Feb-13 | R | 7.97 | 466 | 3.4 | 8:05:00 |
| WQM717 | Snapper | D1 | 8 | 2 | 29.9 | 34.6 | 1952 | 2247 | 16-Feb-13 | R | 7.97 | 466 | 3.4 | 8:05:00 |
| WQM718 | Fitzroy | D0 | 6 | 1 | 29.7 | 34.3 | 1943 | 2213 | 17-Feb-13 | CR | 7.94 | 502 | 3.2 | 8:10:00 |
| WQM718 | Fitzroy | D0 | 6 | 2 | 29.7 | 34.3 | 1944 | 2215 | 17-Feb-13 | CR | 7.94 | 501 | 3.2 | 8:10:00 |
| WQM719 | Fitzroy | D0 | 0 | 1 | 29.7 | 34.2 | 1934 | 2224 | 17-Feb-13 | R | 7.97 | 460 | 3.4 | 9:13:00 |
| WQM719 | Fitzroy | D0 | 0 | 2 | 29.7 | 34.2 | 1936 | 2224 | 17-Feb-13 | R | 7.97 | 464 | 3.3 | 9:13:00 |
| WQM719 | Fitzroy | D2 | 14 | 1 | 29.7 | 34.4 | 1946 | 2237 | 17-Feb-13 | R | 7.97 | 464 | 3.4 | 9:13:00 |
| WQM719 | Fitzroy | D2 | 14 | 2 | 29.7 | 34.4 | 1942 | 2237 | 17-Feb-13 | R | 7.97 | 456 | 3.4 | 9:13:00 |
| WQM720 | High | D0 | 5.5 | 1 | 30.0 | 33.9 | 1920 | 2201 | 17-Feb-13 | CR | 7.96 | 470 | 3.3 | 11:40:00 |
| WQM720 | High | D0 | 5.5 | 2 | 30.0 | 33.9 | 1924 | 2201 | 17-Feb-13 | CR | 7.95 | 481 | 3.2 | 11:40:00 |
| WQM721 | High | D0 | 0 | 1 | 30.0 | 33.5 | 1907 | 2183 | 17-Feb-13 | R | 7.96 | 472 | 3.2 | 12:44:00 |
| WQM721 | High | D0 | 0 | 2 | 30.0 | 33.5 | 1907 | 2183 | 17-Feb-13 | R | 7.96 | 472 | 3.2 | 12:44:00 |
| WQM721 | High | D2 | 20 | 1 | 29.7 | 34.2 | 1940 | 2226 | 17-Feb-13 | R | 7.96 | 470 | 3.3 | 12:44:00 |
| WQM721 | High | D2 | 20 | 2 | 29.7 | 34.2 | 1939 | 2226 | 17-Feb-13 | R | 7.96 | 466 | 3.3 | 12:44:00 |
| WQM722 | Russell | D0 | 6.4 | 1 | 29.6 | 34.5 | 1935 | 2230 | 17-Feb-13 | CR | 7.98 | 451 | 3.4 | 14:10:00 |
| WQM722 | Russell | D0 | 6.4 | 2 | 29.6 | 34.5 | 1936 | 2232 | 17-Feb-13 | CR | 7.98 | 450 | 3.4 | 14:10:00 |
| WQM723 | Russell | D0 | 0 | 1 | 29.6 | 33.9 | 1923 | 2206 | 17-Feb-13 | R | 7.97 | 463 | 3.3 | 15:12:00 |
| WQM723 | Russell | D0 | 0 | 2 | 29.6 | 33.9 | 1924 | 2205 | 17-Feb-13 | R | 7.96 | 465 | 3.3 | 15:12:00 |
| WQM723 | Russell | D3 | 20 | 1 | 29.6 | 34.6 | 1955 | 2244 | 17-Feb-13 | R | 7.96 | 472 | 3.3 | 15:12:00 |
| WQM723 | Russell | D3 | 20 | 2 | 29.6 | 34.6 | 1953 | 2245 | 17-Feb-13 | R | 7.97 | 465 | 3.4 | 15:12:00 |
| WQM726 | Dunk | D0 | 0 | 1 | 29.5 | 31.6 | 1847 | 2070 | 18-Feb-13 | R | 7.91 | 525 | 2.7 | 9:23:00 |
| WQM726 | Dunk | D0 | 0 | 2 | 29.5 | 31.6 | 1848 | 2069 | 18-Feb-13 | R | 7.90 | 532 | 2.7 | 9:23:00 |
| WQM726 | Dunk | D1 | 5 | 1 | 29.8 | 33.7 | 1927 | 2194 | 18-Feb-13 | R | 7.94 | 494 | 3.1 | 9:23:00 |
| WQM726 | Dunk | D1 | 5 | 2 | 29.8 | 33.7 | 1931 | 2196 | 18-Feb-13 | R | 7.94 | 500 | 3.1 | 9:23:00 |
| WQM727 | Dunk | D0 | 5.5 | 1 | 29.8 | 34.1 | 1939 | 2213 | 18-Feb-13 | CR | 7.95 | 491 | 3.2 | 10:30:00 |
| WQM727 | Dunk | D0 | 5.5 | 2 | 29.8 | 34.1 | 1939 | 2213 | 18-Feb-13 | CR | 7.95 | 491 | 3.2 | 10:30:00 |
| WQM730 | Orpheus | D0 | 5.5 | 1 | 29.1 | 34.3 | 1951 | 2238 | 19-Feb-13 | CR | 7.97 | 462 | 3.3 | 8:00:00 |
| WQM730 | Orpheus | D0 | 5.5 | 2 | 29.1 | 34.3 | 1949 | 2237 | 19-Feb-13 | CR | 7.97 | 459 | 3.3 | 8:00:00 |
| WQM731 | Orpheus | D0 | 0 | 1 | 29.1 | 34.3 | 1938 | 2235 | 19-Feb-13 | R | 7.99 | 439 | 3.4 | 8:59:00 |
| WQM731 | Orpheus | D0 | 0 | 2 | 29.1 | 34.3 | 1938 | 2234 | 19-Feb-13 | R | 7.99 | 439 | 3.4 | 8:59:00 |
| WQM731 | Orpheus | D2 | 28 | 1 | 29.1 | 34.5 | 1945 | 2240 | 19-Feb-13 | R | 7.98 | 449 | 3.4 | 8:59:00 |
| WQM731 | Orpheus | D2 | 28 | 2 | 29.1 | 34.5 | 1945 | 2239 | 19-Feb-13 | R | 7.98 | 450 | 3.4 | 8:59:00 |
| WQM732 | Pandora Reef | D0 | 7.2 | 1 | 30.5 | 34.1 | 1970 | 2258 | 19-Feb-13 | CR | 7.95 | 490 | 3.4 | 11:40:00 |
| WQM732 | Pandora Reef | D0 | 7.2 | 2 | 30.5 | 34.1 | 1951 | 2245 | 19-Feb-13 | CR | 7.96 | 472 | 3.4 | 11:40:00 |
| WQM733 | Pandora Reef | D0 | 0 | 1 | 30.5 | 34.0 | 1958 | 2249 | 19-Feb-13 | R | 7.96 | 479 | 3.4 | 12:36:00 |
| WQM733 | Pandora Reef | D0 | 0 | 2 | 30.5 | 34.0 | 1956 | 2250 | 19-Feb-13 | R | 7.97 | 471 | 3.5 | 12:36:00 |
| WQM733 | Pandora Reef | D1 | 10 | 1 | 28.9 | 34.1 | 1957 | 2251 | 19-Feb-13 | R | 7.99 | 445 | 3.4 | 12:36:00 |
| WQM733 | Pandora Reef | D1 | 10 | 2 | 28.9 | 34.1 | 1959 | 2251 | 19-Feb-13 | R | 7.98 | 450 | 3.4 | 12:36:00 |
| WQM735 | Geoffrey Bay | D0 | 6.8 | 1 | 29.4 | 34.2 | 1960 | 2247 | 20-Feb-13 | CR | 7.97 | 469 | 3.4 | 8:10:00 |
| WQM735 | Geoffrey Bay | D0 | 6.8 | 2 | 29.4 | 34.2 | 1960 | 2247 | 20-Feb-13 | CR | 7.97 | 469 | 3.3 | 8:10:00 |
| WQM736 | Geoffrey Bay | D0 | 0 | 1 | 29.4 | 34.0 | 1949 | 2244 | 20-Feb-13 | R | 7.98 | 448 | 3.4 | 9:17:00 |
| WQM736 | Geoffrey Bay | D0 | 0 | 2 | 29.4 | 34.0 | 1952 | 2245 | 20-Feb-13 | R | 7.98 | 453 | 3.4 | 9:17:00 |
| WQM736 | Geoffrey Bay | D1 | 8 | 1 | 28.9 | 34.3 | 1973 | 2258 | 20-Feb-13 | R | 7.97 | 472 | 3.3 | 9:17:00 |
| WQM736 | Geoffrey Bay | D1 | 8 | 2 | 28.9 | 34.3 | 1970 | 2254 | 20-Feb-13 | R | 7.97 | 471 | 3.3 | 9:17:00 |
| WQM737 | Double Cone | D0 | 6.8 | 1 | 28.5 | 34.7 | 1969 | 2257 | 21-Feb-13 | CR | 7.97 | 461 | 3.3 | 8:15:00 |
| WQM737 | Double Cone | D0 | 6.8 | 2 | 28.5 | 34.7 | 1969 | 2255 | 21-Feb-13 | CR | 7.97 | 464 | 3.3 | 8:15:00 |
| WQM738 | Double Cone | D0 | 0 | 1 | 28.5 | 34.7 | 1969 | 2262 | 21-Feb-13 | R | 7.98 | 453 | 3.4 | 10:06:00 |
| WQM738 | Double Cone | D0 | 0 | 2 | 28.5 | 34.7 | 1966 | 2261 | 21-Feb-13 | R | 7.98 | 448 | 3.4 | 10:06:00 |
| WQM738 | Double Cone | D2 | 20 | 1 | 28.3 | 34.7 | 1967 | 2260 | 21-Feb-13 | R | 7.98 | 449 | 3.4 | 10:06:00 |
| WQM738 | Double Cone | D2 | 20 | 2 | 28.3 | 34.7 | 1972 | 2264 | 21-Feb-13 | R | 7.98 | 452 | 3.4 | 10:06:00 |
| WQM739 | Daydream | D0 | 6.2 | 1 | 28.4 | 34.6 | 1962 | 2250 | 21-Feb-13 | CR | 7.98 | 456 | 3.3 | 13:40:00 |
| WQM739 | Daydream | D0 | 6.2 | 2 | 28.4 | 34.6 | 1957 | 2249 | 21-Feb-13 | CR | 7.98 | 446 | 3.4 | 13:40:00 |
| WQM740 | Daydream | D0 | 0 | 1 | 28.4 | 34.6 | 1960 | 2250 | 21-Feb-13 | R | 7.98 | 452 | 3.3 | 11:53:00 |
| WQM740 | Daydream | D0 | 0 | 2 | 28.4 | 34.6 | 1963 | 2253 | 21-Feb-13 | R | 7.98 | 452 | 3.3 | 11:53:00 |
| WQM740 | Daydream | D2 | 22 | 1 | 28.3 | 34.6 | 1966 | 2254 | 21-Feb-13 | R | 7.98 | 455 | 3.3 | 11:53:00 |
| WQM740 | Daydream | D2 | 22 | 2 | 28.3 | 34.6 | 1968 | 2253 | 21-Feb-13 | R | 7.97 | 461 | 3.3 | 11:53:00 |
| WQM741 | Pine | D0 | 8.4 | 1 | 28.2 | 34.6 | 1966 | 2252 | 22-Feb-13 | CR | 7.98 | 458 | 3.3 | 8:10:00 |
| WQM741 | Pine | D0 | 8.4 | 2 | 28.2 | 34.6 | 1965 | 2252 | 22-Feb-13 | CR | 7.98 | 455 | 3.3 | 8:10:00 |
| WQM742 | Pine | D0 | 0 | 1 | 28.2 | 34.6 | 1965 | 2252 | 22-Feb-13 | R | 7.98 | 454 | 3.3 | 9:26:00 |
| WQM742 | Pine | D0 | 0 | 2 | 28.2 | 34.6 | 1966 | 2252 | 22-Feb-13 | R | 7.98 | 458 | 3.3 | 9:26:00 |
| WQM742 | Pine | D2 | 22 | 1 | 28.2 | 34.6 | 1963 | 2253 | 22-Feb-13 | R | 7.98 | 449 | 3.3 | 9:26:00 |
| WQM742 | Pine | D2 | 22 | 2 | 28.2 | 34.6 | 1965 | 2252 | 22-Feb-13 | R | 7.98 | 456 | 3.3 | 9:26:00 |
| WQM744 | Pelican | D0 | 5.1 | 1 | 28.2 | 31.5 | 1851 | 2110 | 23-Feb-13 | CR | 7.99 | 425 | 3.0 | 13:50:00 |
| WQM744 | Pelican | D0 | 5.1 | 2 | 28.2 | 31.5 | 1852 | 2110 | 23-Feb-13 | CR | 7.99 | 425 | 3.0 | 13:50:00 |
| WQM745 | Pelican | D0 | 0 | 1 | 27.8 | 31.4 | 1845 | 2107 | 23-Feb-13 | R | 8.01 | 407 | 3.1 | 15:08:00 |
| WQM745 | Pelican | D0 | 0 | 2 | 27.8 | 31.4 | 1847 | 2107 | 23-Feb-13 | R | 8.00 | 410 | 3.1 | 15:08:00 |
| WQM745 | Pelican | D1 | 7 | 1 | 27.2 | 31.8 | 1862 | 2123 | 23-Feb-13 | R | 8.00 | 412 | 3.0 | 15:08:00 |
| WQM745 | Pelican | D1 | 7 | 2 | 27.2 | 31.8 | 1861 | 2124 | 23-Feb-13 | R | 8.01 | 410 | 3.1 | 15:08:00 |
| WQM746 | Humpy | D0 | 9 | 1 | 27.2 | 33.5 | 1935 | 2203 | 24-Feb-13 | CR | 7.98 | 448 | 3.1 | 8:15:00 |
| WQM746 | Humpy | D0 | 9 | 2 | 27.2 | 33.5 | 1929 | 2203 | 24-Feb-13 | CR | 7.99 | 433 | 3.2 | 8:15:00 |
| WQM747 | Humpy | D0 | 0 | 1 | 27.2 | 31.8 | 1859 | 2119 | 24-Feb-13 | R | 8.00 | 413 | 3.0 | 9:17:00 |
| WQM747 | Humpy | D0 | 0 | 2 | 27.2 | 31.8 | 1861 | 2123 | 24-Feb-13 | R | 8.00 | 411 | 3.1 | 9:17:00 |
| WQM747 | Humpy | D2 | 18 | 1 | 26.5 | 34.0 | 1945 | 2224 | 24-Feb-13 | R | 8.00 | 424 | 3.2 | 9:17:00 |
| WQM747 | Humpy | D2 | 18 | 2 | 26.5 | 34.0 | 1951 | 2227 | 24-Feb-13 | R | 7.99 | 434 | 3.2 | 9:17:00 |
| WQM748 | Barren | D0 | 8 | 1 | 26.8 | 34.0 | 1939 | 2221 | 24-Feb-13 | CR | 8.00 | 423 | 3.2 | 14:30:00 |
| WQM748 | Barren | D0 | 8 | 2 | 26.8 | 34.0 | 1944 | 2226 | 24-Feb-13 | CR | 8.00 | 426 | 3.2 | 14:30:00 |
| WQM749 | Barren | D0 | 0 | 1 | 26.8 | 33.0 | 1893 | 2168 | 24-Feb-13 | R | 8.01 | 406 | 3.2 | 15:29:00 |
| WQM749 | Barren | D0 | 0 | 2 | 26.8 | 33.0 | 1897 | 2172 | 24-Feb-13 | R | 8.01 | 410 | 3.2 | 15:29:00 |
| WQM749 | Barren | D1 | 13 | 1 | 26.5 | 34.0 | 1956 | 2228 | 24-Feb-13 | R | 7.99 | 442 | 3.1 | 15:29:00 |
| WQM749 | Barren | D1 | 13 | 2 | 26.5 | 34.0 | 1956 | 2229 | 24-Feb-13 | R | 7.99 | 440 | 3.1 | 15:29:00 |
| WQM750 | Barren | D0 | 8.9 | 1 | 21.9 | 35.3 | 2007 | 2295 | 13-Jun-13 | CR | 8.06 | 373 | 3.2 | 8:10:00 |
| WQM750 | Barren | D0 | 8.9 | 2 | 21.9 | 35.3 | 2005 | 2295 | 13-Jun-13 | CR | 8.06 | 368 | 3.2 | 8:10:00 |
| WQM751 | Barren | D0 | 0 | 1 | 21.9 | 35.3 | 2002 | 2300 | 13-Jun-13 | R | 8.07 | 358 | 3.3 | 9:27:00 |
| WQM751 | Barren | D0 | 0 | 2 | 21.9 | 35.3 | 1997 | 2301 | 13-Jun-13 | R | 8.08 | 348 | 3.4 | 9:27:00 |
| WQM751 | Barren | D1 | 14 | 1 | 21.7 | 35.2 | 2027 | 2309 | 13-Jun-13 | R | 8.05 | 387 | 3.1 | 9:27:00 |
| WQM751 | Barren | D1 | 14 | 2 | 21.7 | 35.2 | 2029 | 2308 | 13-Jun-13 | R | 8.04 | 390 | 3.1 | 9:27:00 |
| WQM752 | Humpy | D0 | 8.5 | 1 | 22.2 | 35.2 | 2032 | 2303 | 13-Jun-13 | CR | 8.02 | 412 | 3.0 | 11:10:00 |
| WQM752 | Humpy | D0 | 8.5 | 2 | 22.2 | 35.2 | 2002 | 2295 | 13-Jun-13 | CR | 8.06 | 368 | 3.2 | 11:10:00 |
| WQM753 | Humpy | D0 | 0 | 1 | 22.2 | 35.2 | 2031 | 2311 | 13-Jun-13 | R | 8.04 | 399 | 3.1 | 13:07:00 |
| WQM753 | Humpy | D0 | 0 | 2 | 22.2 | 35.2 | 2009 | 2298 | 13-Jun-13 | R | 8.06 | 376 | 3.2 | 13:07:00 |
| WQM753 | Humpy | D2 | 16 | 1 | 21.6 | 35.2 | 2026 | 2305 | 13-Jun-13 | R | 8.05 | 389 | 3.1 | 13:07:00 |
| WQM753 | Humpy | D2 | 16 | 2 | 21.6 | 35.2 | 2031 | 2306 | 13-Jun-13 | R | 8.04 | 396 | 3.1 | 13:07:00 |
| WQM754 | Pelican | D0 | 6.4 | 1 | 22.4 | 34.8 | 2043 | 2311 | 13-Jun-13 | CR | 8.02 | 421 | 3.0 | 14:50:00 |
| WQM754 | Pelican | D0 | 6.4 | 2 | 22.4 | 34.8 | 2021 | 2299 | 13-Jun-13 | CR | 8.04 | 396 | 3.1 | 14:50:00 |
| WQM755 | Pelican | D0 | 0 | 1 | 22.4 | 33.8 | 2041 | 2301 | 13-Jun-13 | R | 8.02 | 423 | 3.0 | 15:49:00 |
| WQM755 | Pelican | D0 | 0 | 2 | 22.4 | 33.8 | 2042 | 2298 | 13-Jun-13 | R | 8.01 | 430 | 2.9 | 15:49:00 |
| WQM755 | Pelican | D1 | 6 | 1 | 21.4 | 34.6 | 2046 | 2310 | 13-Jun-13 | R | 8.03 | 412 | 3.0 | 15:49:00 |
| WQM755 | Pelican | D1 | 6 | 2 | 21.4 | 34.6 | 2047 | 2308 | 13-Jun-13 | R | 8.02 | 417 | 2.9 | 15:49:00 |
| WQM757 | Pine | D0 | 6.4 | 1 | 23.0 | 33.9 | 1983 | 2229 | 15-Jun-13 | CR | 7.99 | 439 | 2.8 | 10:30:00 |
| WQM757 | Pine | D0 | 6.4 | 2 | 23.0 | 33.9 | 1982 | 2231 | 15-Jun-13 | CR | 8.00 | 431 | 2.8 | 10:30:00 |
| WQM758 | Pine | D0 | 0 | 1 | 23.0 | 33.9 | 1963 | 2224 | 15-Jun-13 | R | 8.02 | 405 | 2.9 | 11:38:00 |
| WQM758 | Pine | D0 | 0 | 2 | 23.0 | 33.9 | 1960 | 2224 | 15-Jun-13 | R | 8.03 | 398 | 3.0 | 11:38:00 |
| WQM758 | Pine | D2 | 18 | 1 | 23.0 | 33.9 | 1961 | 2222 | 15-Jun-13 | R | 8.02 | 402 | 2.9 | 11:38:00 |
| WQM758 | Pine | D2 | 18 | 2 | 23.0 | 33.9 | 1983 | 2231 | 15-Jun-13 | R | 8.00 | 435 | 2.8 | 11:38:00 |
| WQM759 | Daydream | D0 | 7 | 1 | 23.0 | 33.9 | 1981 | 2231 | 15-Jun-13 | CR | 8.00 | 430 | 2.8 | 15:00:00 |
| WQM759 | Daydream | D0 | 7 | 2 | 23.0 | 33.9 | 1978 | 2231 | 15-Jun-13 | CR | 8.01 | 423 | 2.9 | 15:00:00 |
| WQM760 | Daydream | D0 | 0 | 1 | 23.0 | 33.8 | 1982 | 2232 | 15-Jun-13 | R | 8.00 | 429 | 2.9 | 13:46:00 |
| WQM760 | Daydream | D0 | 0 | 2 | 23.0 | 33.8 | 1985 | 2234 | 15-Jun-13 | R | 8.00 | 432 | 2.8 | 13:46:00 |
| WQM760 | Daydream | D2 | 22 | 1 | 22.8 | 33.9 | 1988 | 2230 | 15-Jun-13 | R | 7.99 | 445 | 2.7 | 13:46:00 |
| WQM760 | Daydream | D2 | 22 | 2 | 22.8 | 33.9 | 1963 | 2221 | 15-Jun-13 | R | 8.02 | 405 | 2.9 | 13:46:00 |
| WQM761 | Double Cone | D0 | 4.2 | 1 | 22.8 | 33.9 | 1994 | 2222 | 16-Jun-13 | CR | 7.96 | 479 | 2.6 | 8:30:00 |
| WQM761 | Double Cone | D0 | 4.2 | 2 | 22.8 | 33.9 | 1981 | 2209 | 16-Jun-13 | CR | 7.96 | 474 | 2.6 | 8:30:00 |
| WQM762 | Double Cone | D0 | 0 | 1 | 22.8 | 33.8 | 1984 | 2232 | 16-Jun-13 | R | 8.00 | 432 | 2.8 | 10:02:00 |
| WQM762 | Double Cone | D0 | 0 | 2 | 22.8 | 33.8 | 1963 | 2222 | 16-Jun-13 | R | 8.02 | 404 | 2.9 | 10:02:00 |
| WQM762 | Double Cone | D2 | 16 | 1 | 22.9 | 33.8 | 1987 | 2231 | 16-Jun-13 | R | 7.99 | 442 | 2.8 | 10:02:00 |
| WQM762 | Double Cone | D2 | 16 | 2 | 22.9 | 33.8 | 1972 | 2216 | 16-Jun-13 | R | 7.99 | 435 | 2.8 | 10:02:00 |
| WQM764 | Geoffrey Bay | D0 | 5.6 | 1 | 22.7 | 34.4 | 2016 | 2261 | 17-Jun-13 | CR | 7.99 | 452 | 2.8 | 8:30:00 |
| WQM764 | Geoffrey Bay | D0 | 5.6 | 2 | 22.7 | 34.4 | 1999 | 2251 | 17-Jun-13 | CR | 8.00 | 435 | 2.8 | 8:30:00 |
| WQM765 | Geoffrey Bay | D0 | 0 | 1 | 22.7 | 34.4 | 2024 | 2273 | 17-Jun-13 | R | 7.99 | 448 | 2.8 | 9:27:00 |
| WQM765 | Geoffrey Bay | D0 | 0 | 2 | 22.7 | 34.4 | 2007 | 2264 | 17-Jun-13 | R | 8.01 | 427 | 2.9 | 9:27:00 |
| WQM765 | Geoffrey Bay | D1 | 7 | 1 | 22.7 | 34.4 | 2009 | 2266 | 17-Jun-13 | R | 8.01 | 427 | 2.9 | 9:27:00 |
| WQM765 | Geoffrey Bay | D1 | 7 | 2 | 22.7 | 34.4 | 2026 | 2274 | 17-Jun-13 | R | 7.99 | 451 | 2.8 | 9:27:00 |
| WQM766 | Pandora Reef | D0 | 8 | 1 | 23.3 | 34.4 | 1972 | 2244 | 17-Jun-13 | CR | 8.03 | 399 | 3.1 | 13:40:00 |
| WQM766 | Pandora Reef | D0 | 8 | 2 | 23.3 | 34.4 | 1970 | 2247 | 17-Jun-13 | CR | 8.04 | 389 | 3.1 | 13:40:00 |
| WQM767 | Pandora Reef | D0 | 0 | 1 | 23.3 | 34.4 | 1974 | 2248 | 17-Jun-13 | R | 8.03 | 397 | 3.1 | 14:44:00 |
| WQM767 | Pandora Reef | D0 | 0 | 2 | 23.3 | 34.4 | 2000 | 2260 | 17-Jun-13 | R | 8.00 | 431 | 2.9 | 14:44:00 |
| WQM767 | Pandora Reef | D1 | 11 | 1 | 22.8 | 34.4 | 1982 | 2249 | 17-Jun-13 | R | 8.03 | 401 | 3.0 | 14:44:00 |
| WQM767 | Pandora Reef | D1 | 11 | 2 | 22.8 | 34.4 | 1979 | 2249 | 17-Jun-13 | R | 8.03 | 397 | 3.0 | 14:44:00 |
| WQM768 | Orpheus | D0 | 6 | 1 | 23.4 | 34.6 | 1980 | 2252 | 18-Jun-13 | CR | 8.02 | 406 | 3.1 | 8:10:00 |
| WQM768 | Orpheus | D0 | 6 | 2 | 23.4 | 34.6 | 2000 | 2262 | 18-Jun-13 | CR | 8.00 | 431 | 3.0 | 8:10:00 |
| WQM769 | Orpheus | D0 | 0 | 1 | 23.4 | 34.6 | 1996 | 2264 | 18-Jun-13 | R | 8.01 | 418 | 3.0 | 9:02:00 |
| WQM769 | Orpheus | D0 | 0 | 2 | 23.4 | 34.6 | 1973 | 2253 | 18-Jun-13 | R | 8.04 | 389 | 3.1 | 9:02:00 |
| WQM769 | Orpheus | D2 | 28 | 1 | 23.5 | 34.7 | 2001 | 2263 | 18-Jun-13 | R | 8.00 | 433 | 3.0 | 9:02:00 |
| WQM769 | Orpheus | D2 | 28 | 2 | 23.5 | 34.7 | 2001 | 2267 | 18-Jun-13 | R | 8.01 | 425 | 3.0 | 9:02:00 |
| WQM778 | Snapper | D0 | 6.5 | 1 | 24.1 | 34.1 | 1955 | 2209 | 20-Jun-13 | CR | 7.99 | 432 | 2.9 | 9:40:00 |
| WQM778 | Snapper | D0 | 6.5 | 2 | 24.1 | 34.1 | 1977 | 2223 | 20-Jun-13 | CR | 7.98 | 457 | 2.8 | 9:40:00 |
| WQM779 | Snapper | D0 | 0 | 1 | 24.1 | 34.1 | 1949 | 2220 | 20-Jun-13 | R | 8.02 | 402 | 3.1 | 10:59:00 |
| WQM779 | Snapper | D0 | 0 | 2 | 24.1 | 34.1 | 1950 | 2220 | 20-Jun-13 | R | 8.02 | 403 | 3.1 | 10:59:00 |
| WQM779 | Snapper | D1 | 7 | 1 | 24.1 | 34.1 | 1982 | 2239 | 20-Jun-13 | R | 7.99 | 440 | 2.9 | 10:59:00 |
| WQM779 | Snapper | D1 | 7 | 2 | 24.1 | 34.1 | 1978 | 2236 | 20-Jun-13 | R | 7.99 | 437 | 2.9 | 10:59:00 |
| WQM780 | Fitzroy | D0 | 6.2 | 1 | 24.1 | 34.3 | 1963 | 2230 | 21-Jun-13 | CR | 8.01 | 416 | 3.0 | 8:20:00 |
| WQM780 | Fitzroy | D0 | 6.2 | 2 | 24.1 | 34.3 | 1964 | 2229 | 21-Jun-13 | CR | 8.01 | 421 | 3.0 | 8:20:00 |
| WQM781 | Fitzroy | D0 | 0 | 1 | 23.5 | 34.2 | 1965 | 2225 | 21-Jun-13 | R | 8.01 | 418 | 2.9 | 9:10:00 |
| WQM781 | Fitzroy | D0 | 0 | 2 | 23.5 | 34.2 | 1959 | 2229 | 21-Jun-13 | R | 8.03 | 398 | 3.0 | 9:10:00 |
| WQM781 | Fitzroy | D2 | 14 | 1 | 23.5 | 34.3 | 1964 | 2233 | 21-Jun-13 | R | 8.02 | 404 | 3.0 | 9:10:00 |
| WQM781 | Fitzroy | D2 | 14 | 2 | 23.5 | 34.3 | 1986 | 2239 | 21-Jun-13 | R | 7.99 | 441 | 2.9 | 9:10:00 |
| WQM782 | High | D0 | 4 | 1 | 23.4 | 34.0 | 1950 | 2210 | 21-Jun-13 | CR | 8.01 | 410 | 2.9 | 11:40:00 |
| WQM782 | High | D0 | 4 | 2 | 23.4 | 34.0 | 1916 | 2197 | 21-Jun-13 | CR | 8.05 | 363 | 3.1 | 11:40:00 |
| WQM783 | High | D0 | 0 | 1 | 23.4 | 33.9 | 1945 | 2216 | 21-Jun-13 | R | 8.03 | 389 | 3.0 | 12:51:00 |
| WQM783 | High | D0 | 0 | 2 | 23.4 | 33.9 | 1943 | 2218 | 21-Jun-13 | R | 8.04 | 381 | 3.1 | 12:51:00 |
| WQM783 | High | D2 | 20 | 1 | 23.4 | 33.9 | 1950 | 2222 | 21-Jun-13 | R | 8.03 | 388 | 3.0 | 12:51:00 |
| WQM783 | High | D2 | 20 | 2 | 23.4 | 33.9 | 1951 | 2222 | 21-Jun-13 | R | 8.03 | 389 | 3.0 | 12:51:00 |
| WQM784 | Russell | D0 | 5.2 | 1 | 23.6 | 34.4 | 1958 | 2233 | 21-Jun-13 | CR | 8.03 | 393 | 3.1 | 14:20:00 |
| WQM784 | Russell | D0 | 5.2 | 2 | 23.6 | 34.4 | 1961 | 2236 | 21-Jun-13 | CR | 8.03 | 394 | 3.1 | 14:20:00 |
| WQM785 | Russell | D0 | 0 | 1 | 23.6 | 34.4 | 1964 | 2239 | 21-Jun-13 | R | 8.03 | 395 | 3.1 | 15:19:00 |
| WQM785 | Russell | D0 | 0 | 2 | 23.6 | 34.4 | 1963 | 2237 | 21-Jun-13 | R | 8.03 | 397 | 3.1 | 15:19:00 |
| WQM785 | Russell | D3 | 20 | 1 | 23.6 | 34.4 | 1963 | 2239 | 21-Jun-13 | R | 8.03 | 393 | 3.1 | 15:19:00 |
| WQM785 | Russell | D3 | 20 | 2 | 23.6 | 34.4 | 1969 | 2240 | 21-Jun-13 | R | 8.02 | 405 | 3.0 | 15:19:00 |
| WQM788 | Dunk | D0 | 0 | 1 | 22.7 | 32.4 | 1906 | 2137 | 22-Jun-13 | R | 8.00 | 419 | 2.6 | 9:51:00 |
| WQM788 | Dunk | D0 | 0 | 2 | 22.7 | 32.4 | 1901 | 2140 | 22-Jun-13 | R | 8.02 | 399 | 2.7 | 9:51:00 |
| WQM788 | Dunk | D1 | 5 | 1 | 23.1 | 33.3 | 1940 | 2189 | 22-Jun-13 | R | 8.01 | 412 | 2.8 | 9:51:00 |
| WQM788 | Dunk | D1 | 5 | 2 | 23.1 | 33.3 | 1937 | 2192 | 22-Jun-13 | R | 8.02 | 400 | 2.9 | 9:51:00 |
| WQM789 | Dunk | D0 | 5 | 1 | 23.1 | 33.6 | 1942 | 2197 | 22-Jun-13 | CR | 8.02 | 405 | 2.9 | 10:50:00 |
| WQM789 | Dunk | D0 | 5 | 2 | 23.1 | 33.6 | 1946 | 2196 | 22-Jun-13 | CR | 8.01 | 417 | 2.8 | 10:50:00 |

Supplementary Table 2. Historic water chemistry data from inshore (Bowling Green Bay, Pandora Reef), midshelf (Rib Reef, Davies Reef) and outer shelf reefs (Myrmidon Reef) of the Great Barrier Reef. Samples are the first samples from cross-reef transects, thus close to the windward reef edge and presumed under little influence of reef metabolism. Samples in Cape Bowling Green are not associated with reefs, but represent inshore water close to the coastline. Methods are the same as described in [[1](#_ENREF_1),[2](#_ENREF_2)].

| Run | Location | Date | Replicate | Salinity | Temperature (°C) | Ta  (μmol kg ^-1^) | pH [NBS] |
| --- | --- | --- | --- | --- | --- | --- | --- |
| 228 | Myrmidon Rf. | 7/12/1983 | 1 | 35.48 | 28.2 | 2436 | 8.239 |
| 228 |  | 7/12/1983 | 2 | 35.48 | 28.3 | 2417 | 8.25 |
| 229 |  | 7/12/1983 | 1 | 35.48 | 28.3 | 2420 | 8.24 |
| 229 |  | 7/12/1983 | 2 | 35.48 | 28.4 | 2412 | 8.25 |
| 255 | Rib Rf. | 10/02/1984 | 1 | 35.3 | 27.1 | 2412 | 8.217 |
| 255 |  | 10/02/1984 | 2 | 35.3 | 27.4 | 2401 | 8.231 |
| 268 | Bowling Green Bay | 29/02/1984 | 1 | 32.06 | 27.9 | 2266 | 8.261 |
| 268 |  | 29/02/1984 | 2 | 32.65 | 28.1 | 2291 | 8.267 |
| 275 |  | 1/03/1984 | 1 | 32.02 | 28.1 | 2278 | 8.282 |
| 275 |  | 1/03/1984 | 2 | 32.35 | 28.2 | 2288 | 8.279 |
| 289 | Pandora Rf. | 4/04/1984 | 1 | 35.04 | 27.3 | 2395 | 8.229 |
| 289 |  | 4/04/1984 | 2 | 35.05 | 27.3 | 2391 | 8.244 |
| 290 |  | 4/04/1984 | 1 | 35 | 27.3 | 2393 | 8.231 |
| 290 |  | 4/04/1984 | 2 | 35.01 | 27.3 | 2387 | 8.25 |
| 297 |  | 5/04/1984 | 1 | 35.28 | 26.6 | 2386 | 8.199 |
| 297 |  | 5/04/1984 | 2 | 35.25 | 26.4 | 2388 | 8.201 |
| 304 |  | 6/04/1984 | 1 | 35.27 | 26.9 | 2402 | 8.24 |
| 304 |  | 6/04/1984 | 2 | 35.28 | 27 | 2401 | 8.27 |
| 20 | Davies Rf. | 25/11/1982 | 1 | 35.4 | 25.5 | 2381 | 8.235 |
| 20 |  | 25/11/1982 | 2 | 35.46 | 25.6 | 2365 | 8.241 |
| 46 |  | 14/01/1983 | 1 | 35.29 | 28.5 | 2346 | 8.223 |
| 46 |  | 14/01/1983 | 2 | 35.28 | 28.6 | 2347 | 8.226 |
| 375 | Myrmidon Rf. | 7/07/1984 | 1 | 35.7 | 22.7 | 2393 | 8.209 |
| 375 |  | 7/07/1984 | 2 | 35.7 | 23 | 2400 | 8.223 |
| 398 | Pandorra Rf. | 10/07/1984 | 1 | 36.06 | 21 | 2368 | 8.24 |
| 398 |  | 10/07/1984 | 2 | 36.11 | 20.9 | 2388 | 8.268 |
| 408 | Rib Rf. | 1/09/1984 | 1 | 35.74 | 23.5 | 2371 | 8.245 |
| 408 |  | 1/09/1984 | 2 | 35.74 | 23.6 | 2351 | 8.246 |
| 434 | Pandorra Rf. | 5/09/1984 | 1 | 35.71 | 24 | 2378 | 8.262 |
| 434 |  | 5/09/1984 | 2 | 35.69 | 24 | 2357 | 8.286 |
| 446 | Davies Rf. | 1/08/1984 | 1 | 35.66 | 22.4 | 2384 | 8.294 |
| 446 |  | 1/08/1984 | 2 | 35.68 | 22.7 | 2390 | 8.283 |

**Supplementary Literature**

1. Barnes D, Devereux M (1984) Productivity and calcification on a coral reef: a survey using pH and oxygen electrode techniques. Journal of Experimental Marine Biology and Ecology 79: 213-231.

2. Barnes D (1983) Profiling coral reef productivity and calcification using pH and oxygen electrodes. Journal of Experimental Marine Biology and Ecology 66: 149-161.
